# ISWI1 complex proteins facilitate developmental genome editing in *Paramecium*

**DOI:** 10.1101/gr.278402.123

**Published:** 2025-01

**Authors:** Aditi Singh, Lilia Häußermann, Christiane Emmerich, Emily Nischwitz, Brandon K.B. Seah, Falk Butter, Mariusz Nowacki, Estienne C. Swart

**Affiliations:** 1 Max Planck Institute for Biology, 72076 Tübingen, Germany;; 2 Institute of Molecular Biology, 55128 Mainz, Germany;; 3 Institute of Molecular Virology and Cell Biology (IMVZ), Friedrich Loeffler Institut, 17493 Greifswald, Germany;; 4 Institute of Cell Biology, University of Bern, 3012 Bern, Switzerland

## Abstract

One of the most extensive forms of natural genome editing occurs in ciliates, a group of microbial eukaryotes. Ciliate germline and somatic genomes are contained in distinct nuclei within the same cell. During the massive reorganization process of somatic genome development, ciliates eliminate tens of thousands of DNA sequences from a germline genome copy. Recently, we showed that the chromatin remodeler ISWI1 is required for somatic genome development in the ciliate *Paramecium tetraurelia*. Here, we describe two high similarity paralogous proteins, ICOPa and ICOPb, essential for their genome editing. ICOPa and ICOPb are highly divergent from known proteins; the only domain detected showed distant homology with the WSD (WHIM2 + WHIM3) motif. We show that both ICOPa and ICOPb interact with the chromatin remodeler ISWI1. Upon *ICOP* knockdown, changes in alternative DNA excision boundaries and nucleosome densities are similar to those observed for *ISWI1* knockdown. We thus propose that a complex comprising ISWI1 and either or both ICOPa and ICOPb are needed for *Paramecium*’s precise genome editing.

Chromatin's underlying subunit, the nucleosome, comprises ∼146 bp of DNA wrapped around an octamer of histone proteins (Kornberg 1977). The presence of a nucleosome alters DNA's geometry and physically shields it, affecting interactions with other DNA-binding proteins (Piña et al. 1990; Pryciak and Varmus 1992; Morgunova and Taipale 2021). The nucleosome thus participates in and regulates numerous molecular processes (Campos and Reinberg 2009; Bai and Morozov 2010; Alabert and Groth 2012; Price and D'Andrea 2013).

Nucleosomes can be moved, ejected, or reconstructed with alternative histone variants by four families of ATP-dependent chromatin remodelers (Clapier and Cairns 2009). The imitation switch (ISWI) family of chromatin remodelers forms several complexes capable of nucleosome sliding (Längst et al. 1999) in different organisms, each serving a distinct role. ISWI contains an N-terminal SNF2 ATPase domain that provides energy to move the nucleosome (Li et al. 2019). The C-terminal HAND-SANT-SLIDE (HSS) domain is essential for substrate recognition (Grüne et al. 2003). ISWI complex partners determine the context of the complex activity and alter its remodeling efficiency (Längst et al. 1999; Toto et al. 2014). ISWI complexes have been shown to regulate DNA replication, transcription, DNA repair, and V(D)J cleavage of polynucleosomal DNA (Patenge et al. 2004; Clapier and Cairns 2009; Aydin et al. 2014).

Like other ciliates, *Paramecium* has distinct nuclei: germline micronuclei (MICs) and the somatic macronucleus (MAC). MICs produce gametic nuclei that form a diploid zygotic nucleus, which generates new MICs and MACs. The zygotic genome developing into a new MAC genome undergoes massive editing, excising thousands of germline-limited sequences, and also amplification to a high polyploidy (about 800n) (Drews et al. 2022a; Zangarelli et al. 2022). *Paramecium*’s internal eliminated sequences (IESs) are distributed throughout the germline genome, including coding sequences (Arnaiz et al. 2012). IES removal thus requires precise excision and DNA repair (Kapusta et al. 2011; Dubois et al. 2012).

Each of *Paramecium*’s 45,000 unique IESs is flanked by conserved 5′-TA-3′ dinucleotides, which are part of a less well conserved ∼5 bp terminal inverted repeat (Klobutcher and Herrick 1995; Arnaiz et al. 2012; Bischerour et al. 2018). PiggyMAC (PGM), a domesticated PiggyBac transposase (Baudry et al. 2009; Bischerour et al. 2018), is responsible for the excision of IESs and other germline-specific DNA. The IES length distribution monotonically declines with a characteristic 10/11 bp periodicity, except for a ∼34–44 bp “forbidden” peak, in which IESs appear largely absent, prevented by DNA's topological constraints and necessity of proper PGM subunit orientation for interaction (Arnaiz et al. 2012). IESs lack sufficient motifs for precise recognition and excision necessitating additional molecules for their removal.

*Paramecium* germline-limited sequences are thought to be targeted by two small noncoding RNA classes: scnRNAs and iesRNAs. scnRNAs are produced by Dicer-like proteins Dcl2 and Dcl3 in the MICs and loaded on Piwi proteins Ptiwi01/09 and transported to the old MAC in which “scanning” is thought to subtract non-IES-matching molecules (Lepère et al. 2009; Bouhouche et al. 2011; Sandoval et al. 2014). In the new MAC noncoding transcripts produced by RNA polymerase II with distinct, nucleus-specific TFIIS4 subunits are proposed to bind to scnRNAs and facilitate IES targeting (Maliszewska-Olejniczak et al. 2015). iesRNAs, produced by Dcl5 and Ptiwi10/11 proteins, are proposed to form a positive feedback loop that efficiently excises most IES copies after IES excision onset (Sandoval et al. 2014; Furrer et al. 2017). As a general trend, shorter IESs tend to be older and primarily iesRNA and scnRNA independent, whereas younger, longer IESs require these and other molecules for excision (Sellis et al. 2021). Ptiwi01/09 was recently also suggested to interact with Polycomb repressive complex 2 (PRC2), which represses transposable elements (Miró-Pina et al. 2022; Wang et al. 2022), and also with ISWI1, assisting precise IES excision (Singh et al. 2022).

We recently showed that an ISWI homolog, ISWI1, is required for precise genome editing in *Paramecium tetraurelia* (henceforth, *Paramecium*) (Singh et al. 2022). ISWI1 depletion is lethal, leading to two distinct errors: (1) failure of excision of numerous IESs and (2) excision of IESs at alternative TA boundaries (Singh et al. 2022). In the latter case, excision precision was proposed to be compromised by inappropriate nucleosome positioning. A distinctive characteristic of ISWI1 depletion is substantial alternative “forbidden”-length IES excision. Here, we identified and investigated the contribution to IES excision of ISWI1 complex subunits.

## Results

### Identifying putative components of the ISWI1 complex

Previously, we performed coimmunoprecipitation (co-IP) of 3XFLAG-HA-tagged ISWI1 (Singh et al. 2022). After ISWI1, the most abundant protein candidate detected by protein mass spectrometry (MS), with more than a fivefold enrichment in peptides identified relative to the input, is a 779-amino-acid-long uncharacterized protein (ParameciumDB identifier: PTET.51.1.P0440186). An ohnolog of this protein (PTET.51.1.P0180124 783 amino acids long; 92.04% amino acid sequence identity) is also present in the subset of peptides identified as unique to ISWI1-IP replicates in the same MS data set (Singh et al. 2022). Based on their properties and the experiments reported below, we named our putative interacting candidate ISWI1 Complex Protein a (ICOPa; PTET.51.1.P0440186) and its closely related ohnolog ISWI1 Complex Protein b (ICOPb; PTET.51.1.P0180124), respectively.

**Figure 1.**
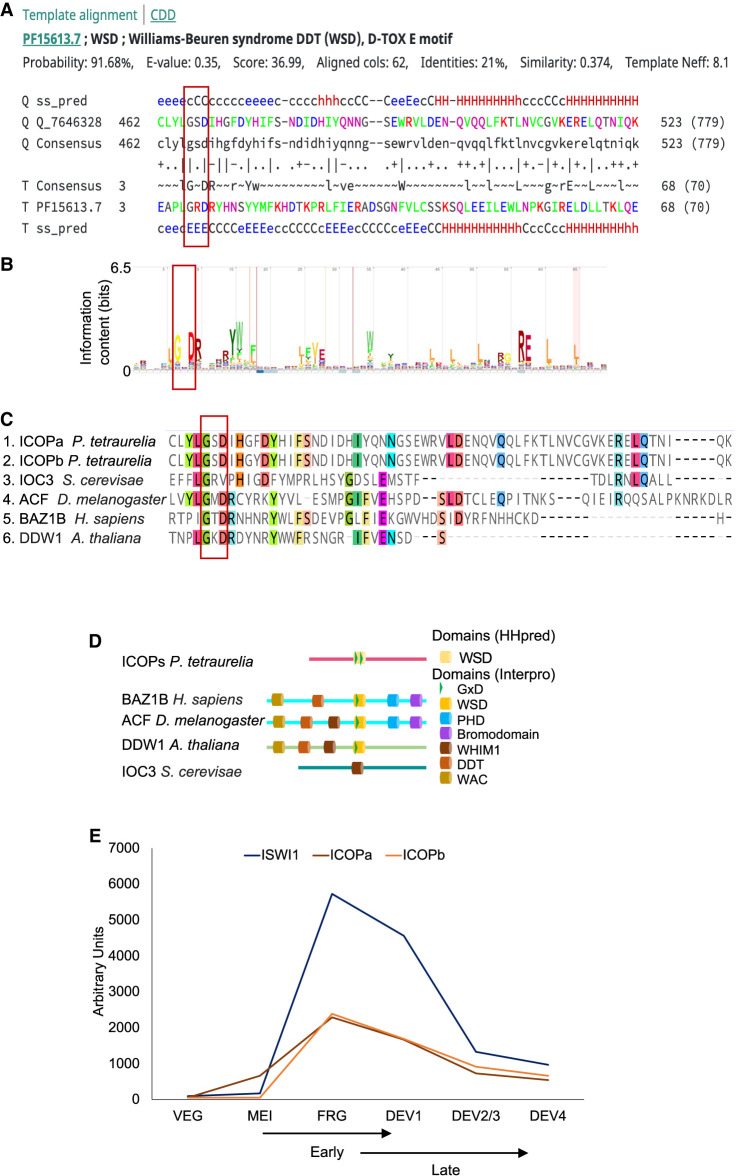
Identification of ISWI complex proteins (ICOPs). (*A*) Template alignment generated by HHpred analysis of ICOPa showing a 91.68% probability match (*E*-value = 0.35) with Williams–Beuren syndrome DDT (WSD) or D-TOX E motif. The conserved GxD signature is highlighted with a red bar. (Q) Query (ICOPa), (ss_pred) secondary structure prediction, and (T) template. For a more detailed output format description, please consult https://toolkit.tuebingen.mpg.de/tools/hhpred. (*B*) Signature of Pfam model PF15613 from InterPro. (*C*) Multiple sequence alignment of ICOPa and ICOPb WSD motif with WSD motif–containing protein regions from other organisms, including ISWI complex. (Ioc3) ISWI one complex protein 3 in yeast, (ACF) ATP-dependent chromatin assembly factor large subunit (Acf) from *D. melanogaster*, (BAZ1B) human tyrosine-protein kinase BAZ1B, and (DDW1) DDT domain-containing protein in *A. thaliana*. Red box indicates GxD signature; highlighted amino acids with ≥50% of residues identical to the consensus residue. (*D*) Domain architecture comparison of ICOPs with ISWI1 complex proteins with WSD motifs. (*E*) mRNA expression profile (arbitrary units) of ICOPa and ICOPb in comparison to ISWI1 during autogamy based on data from ParameciumDB (Arnaiz et al. 2020). (VEG) vegetative, (MEI) the stage at which MICs undergo meiosis and maternal macronucleus (MAC) begins to fragment, (FRG) ∼50% of cells with fragmented maternal MAC, (DEV1) the earliest stage with visible developing MACs (anlage), (DEV2/3) most cells with macronuclear anlage, and (DEV4) most cells with distinct anlage. In this paper, we consider “early” development to be MEI and FRG (T6–T10) and “late” development to be DEV1 and DEV2/3 (T12–T16).

We checked if the candidate proteins have homologs that form ISWI complexes in other organisms (Dirscherl and Krebs 2004). Because HMMER3 Pfam database searches failed to identify any domain (Finn et al. 2003), we searched for more distantly associated domains using HHpred (Zimmermann et al. 2018). HHpred generates a hidden Markov model (HMM) for the query using the iterative search and alignment functionality provided by HHblits (Remmert et al. 2012). The HHpred results indicated a probability of 91.68% for the “D-TOX E motif, Williams–Beuren syndrome DDT (WSD) motif” (Pfam model PF15613; 65 amino acids long, spanning almost the complete model length) (Fig. 1A,B). This domain corresponds to the entire WHIM2 and half of the succeeding WHIM3, that is, two of three “motifs” in a series of so-called WHIM motif proteins (Aravind and Iyer 2012).

The ICOPa and ICOPb proteins had no other detectable domains but contained three amino acid residues, called the GxD signature (Fig. 1A–C; Aravind and Iyer 2012), within the WSD motif. The WSD motif is known to interact with linker DNA and the SLIDE domain in ISWI proteins (Mukherjee et al. 2009; Yamada et al. 2011; Aravind and Iyer 2012). Proteins with WHIM motifs often have multiple domain architectures (Aravind and Iyer 2012). Public databases like Pfam may annotate proteins as single-domain despite having other domains owing to limited detection sensitivity. For example, IOC3 in yeast (UniProt identifier: P43596) is annotated with WHIM1 alone (Fig. 1D) but also has WHIM2 and WHIM3 (Aravind and Iyer 2012).

*ICOPa* and *ICOPb* gene expression is upregulated during autogamy with a profile similar to that of *ISWI1*’s (Fig. 1E). Furthermore, phylogenetic analysis of proteins with the WSD motif suggests that ICOPa and ICOPb are highly divergent relative to other WSD motif–containing proteins (Supplemental Fig. S1). As shown subsequently, the ICOP ohnologs appeared functionally equivalent.

### ICOP proteins localize to the developing MACs during autogamy

**Figure 2.**
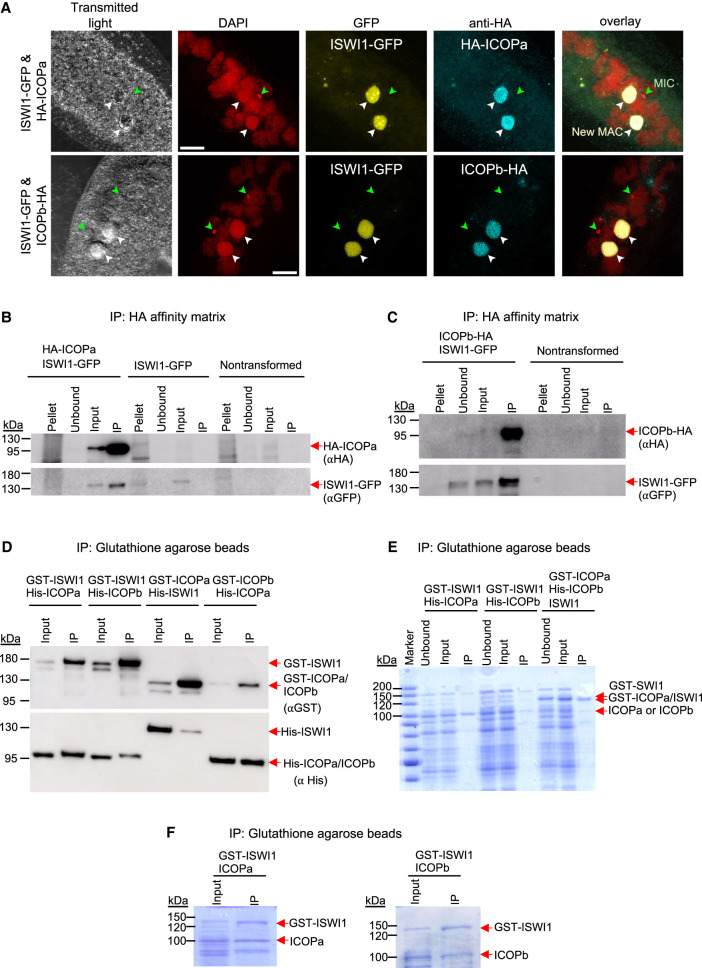
Interaction of ICOPa and ICOPb with ISWI1 in new MACs. (*A*) Confocal fluorescence microscopy images of HA-ICOPa, ICOPb-HA, and ISWI1-GFP localization: maximum intensity projections of *z*-planes. Samples were collected when ∼90% of cells had visible anlagen. Red indicates DAPI; yellow, GFP; cyan, HA; green arrow, MIC; and white arrow, new MAC. All channels were optimized individually for the best visual representation. DAPI channel of ICOPb-HA: Gamma factor = 0.8. Scale bar = 10 µm. (*B*,*C*) Western blot; coimmunoprecipitation (co-IP) of HA-ICOPa/ISWI1-GFP and ICOPb-HA/ISWI1-GFP in *Paramecium*. Controls are nontransformed and ISWI1-GFP-transformed. HA-ICOPa/ICOPb-HA: 94 KDa; ISWI1-GFP: 147 KDa. (*D*–*F*) Co-IP after *E. coli* expression and pulldown. (*D*) Western blot; (*E*,*F*) Coomassie staining. (*D*–*F*) GST-ISWI: 147 kDa; His-ISWI1: 122 kDa; His-ICOPa and His-ICOPb: 95 kDa; GST-ICOPa/ICOPb: 119 kDa; untagged ISWI1: 120 kDa; and untagged ICOPa and ICOPb: 93 kDa.

We cotransformed paramecia with either N-terminally tagged HA-ICOPa or C-terminally tagged ICOPb-HA with ISWI1-GFP to check ICOP localization. Similar to ISWI1 (Singh et al. 2022), ICOPs localized exclusively to the developing MACs during autogamy (Fig. 2A; Supplemental Fig. S2A). We observed no growth defects in the cotransformed cells during vegetative growth or in the F_1_ progeny (Supplemental Fig. S3A). The ICOP paralog localization thus suggests they function at the same stage as ISWI1 during new MAC development.

### ISWI1 and ICOP paralogs form a complex in vivo during autogamy

Using the cotransformed HA-ICOPa/ISWI1-GFP or ICOPb-HA/ISWI1-GFP lysates, we performed reciprocal co-IPs to assess ICOPa and ICOPb interactions with ISWI1. As controls, lysates of nontransformed (wild type [WT]) and ISWI1-GFP-transformed cells were used. WT cells showed no protein pulldown signal with either HA- or GFP-conjugated beads (Fig. 2B,C; Supplemental Fig. S3B). The ISWI1-GFP signal was detected only in the “input” fraction when using the HA-conjugated beads (Fig 2B, lower panel) in ISWI1-GFP transformants. ISWI1-GFP was successfully copurified with HA-ICOPa or ICOPb-HA from the cotransformed cell lysates (Fig. 2B,C; Supplemental Fig. S3B). Co-IPs with ISWI1-GFP, HA-ICOPa, and ICOPb-HA single transformants were further analyzed using protein MS (Supplemental Fig. S3C). ISWI1 was among the most highly enriched proteins, along with either or both of the ICOPs (Supplemental Fig. S3D). Therefore, we conclude that both ICOP paralogs can interact with ISWI1 in *Paramecium*.

### Assessment of GxD signature requirement for ISWI1–ICOP interaction

We tested whether ICOPa and ICOPb could bind directly to ISWI1 by coexpressing them in *Escherichia coli*. GST or His N-terminal fusion proteins or untagged proteins were used for the pulldown. Pulldown specificity was validated using glutathione agarose (GST) or nickel-IMAC agarose (Ni_2_ + NTA) beads. Unspecific binding or cross-reactivity of tagged proteins in the IP fraction of the pulldowns was not observed (Supplemental Fig. S3E–G). ISWI1, ICOPa, and ICOPb were coexpressed in different combinations to perform pulldowns using GST beads. The expected protein interactions were observed in all the pulldown combinations tested (Fig. 2D–F).

**Figure 3.**
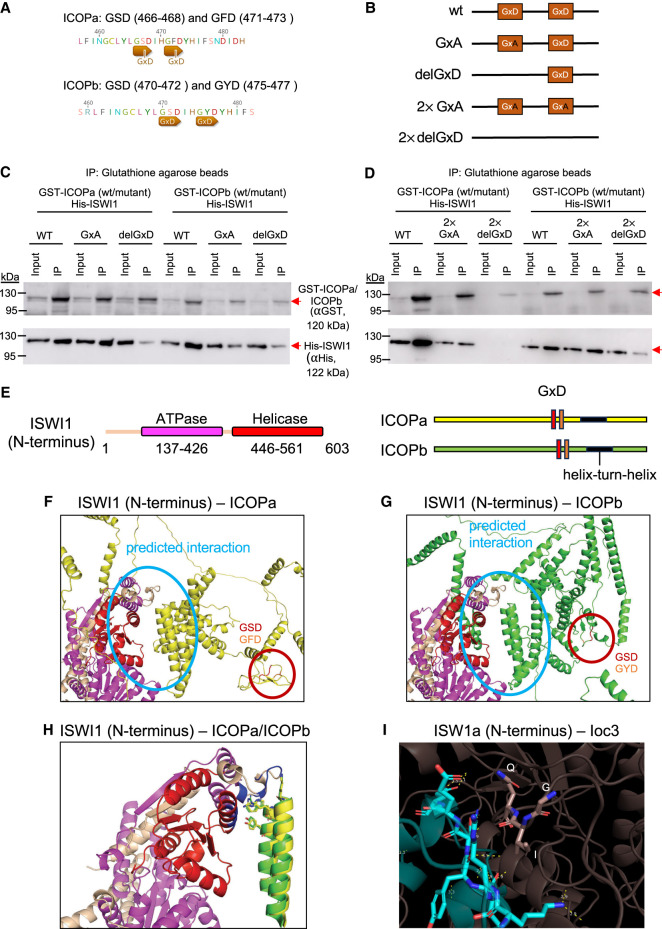
Investigation of the GxD signature in ICOP/ISWI1 interaction. (*A*) Screenshots from Geneious Prime (version 2023.1.1) showing the GxD signature in ICOPa and ICOPb. (*B*) Schematic representation of GxD mutants generated. (*C*,*D*) Western blot of co-IP of GST-ICOP GxD mutants and His-ISWI1 overexpressed in *E*. *coli* probed with anti-GST and anti-His antibodies. GST-ICOP wild-type is a control. (*E*) Schematic representation of the sequences used for complex predictions in *F* and *G*. (*F*–*H*) Structure prediction of multimers (ISWI1 N terminus [residues 1–603] with ICOPa or ICOPb) with AlphaFold (version 2.2.0). Yellow indicates ICOPa; green, ICOPb; red, GSD signature; orange, GFD/GYD signature; wheat, ISWI1; magenta, ISWI1 ATPase domain; and red, ISWI1 helicase domain. (*F*,*G*) ISWI1–ICOPa and ISWI1–ICOPb interaction, respectively. Predicted interaction interface and GxD signatures are circled. (*H*) ISWI1 N terminus with interacting helices of ICOP paralogs (ICOPa: residues 556–597; ICOPb: residues 560–603). Blue indicates proximate residues on ISWI1. Proximate residues of ICOPs are shown as sticks. (*I*) GxD signature in the published crystal structure (PDB accession number 2Y9Y). ISW1a (del_ATPase; cyan) and Ioc3 (WHIM containing protein; dark salmon) from yeast. GxD signature (GIQ in Ioc3) and spatially close residues in ISW1a are shown as sticks. Yellow indicates polar contacts between the proteins.

Because the GxD signature in WSD motif–containing proteins was proposed to mediate interactions with ISWI1 in diverse eukaryotes (Aravind and Iyer 2012), we assessed whether this signature is needed to form the ISWI1–ICOP complex. ICOPa and ICOPb have two GxDs (Fig. 3A). As aspartate was proposed to drive the interaction in the GxD signature (Aravind and Iyer 2012), ICOP mutants with a D-to-A substitution (GxA mutants) were generated. In addition, mutants with the complete GxD deletion (delGxD mutants) were also generated (Fig. 3B). Both mutants coimmunoprecipitated with His-ISWI1 (Fig. 3C,D). A 2× del ICOPa mutant (GSD and GFD removed) pulldown was inefficient (Fig. 3D), barring which His-ISWI1 copurified with all the other ICOP mutants. Therefore, we found no evidence that ISWI1–ICOP interaction requires a GxD signature.

We then explored ISWI1–ICOP interaction using AlphaFold2. ISWI1's predicted structure was of high confidence, and its domains were similar to published structures of yeast ISWI (Supplemental Fig. S4A,B; Yamada et al. 2011; Yan et al. 2019). However, ICOP structure predictions were of low confidence (Supplemental Fig. S4B), likely because of their high divergence compared with other WSD proteins that generated a less informative multiple sequence alignment for structure prediction. We detected large interaction interfaces between ISWI1 and the ICOPs using AlphaFold version 2.3.0 in all the tested combinations. In contrast, AlphaFold2 version 2.2.0 predicted an interaction of ICOPs only with the ISWI1 N terminus (residues 1–603, including the ATPase domain but not the HSS domain) (Fig. 3E–H; Supplemental Table S1). In these models, the ICOPs bound with a defined helix–loop–helix motif (ICOPa: residues 556–597; ICOPb: residues 560–603) (Fig. 3H). Irrespective of the AlphaFold2 version, neither of the GxD signatures was predicted to participate in the interaction (Fig. 3F,G; Supplemental Table S1). Ioc3, a WSD motif–containing ISWI complex protein in yeast, binds to ISW1a C terminus (Yamada et al. 2011; Aravind and Iyer 2012) without any polar interactions between the GxD signature of its WSD and ISW1a (Fig. 3I). Hence, the GxD signature does not appear to be necessary for ISWI1–ICOP interaction.

### *ICOPa/b* knockdown affects cell survival and genome editing

**Figure 4.**
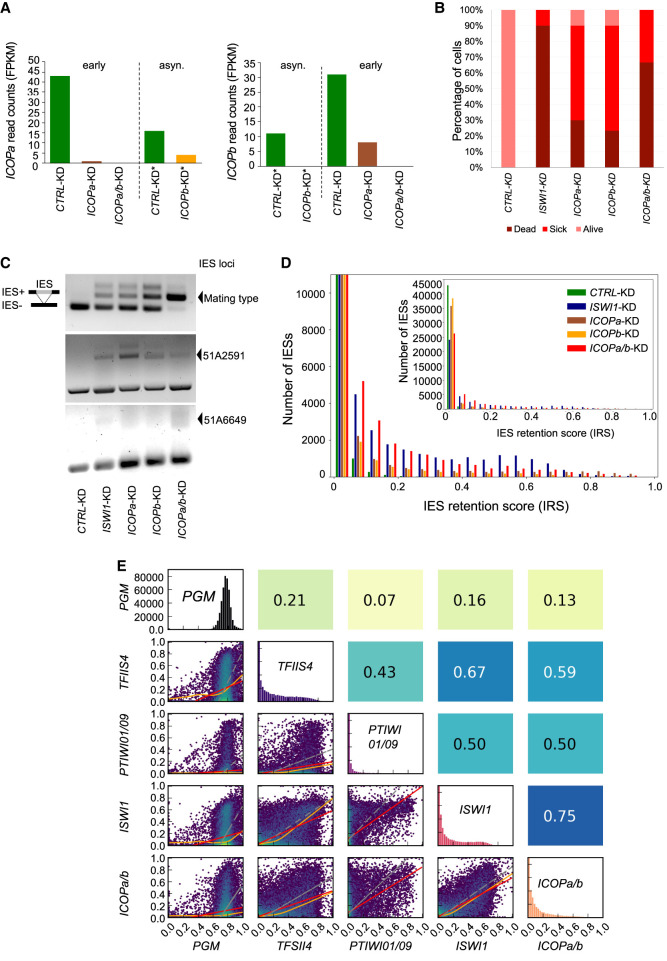
Effects of *ICOP* knockdowns (KDs) on DNA excision. (*A*) mRNA expression levels in fragments per kilobase per million mapped reads (FPKM) compared between *ICOPa* and *ICOPb* KDs for transcripts early in development (40% old MAC fragmentation) or asynchronous (asyn.) cultures (*) in which new MACs were observed in 20% of cells but the majority of the cells were in early development. *ICOPa/b* indicates cosilencing of *ICOPa* and *ICOPb*. (*B*) Survival of recovered postautogamous KD cells followed for several vegetative divisions. Alive (pink): four divisions per day; sick (red): fewer than three divisions per day; dead (cayenne): no cells. (*C*) Retention of mating type, 51A2591 and 51A6649 IESs. *ISWI1*-KD: positive control. Retained IESs (IES+) result in a larger amplicon. (*D*) Genome-wide IES retention scores (IRS) across ∼45,000 IESs in different KDs. (*E*) Correlation of IRSs among KDs. Diagonal indicates IRS distributions of individual KDs; below diagonal, correlation graphs of pairwise comparisons; above diagonal, corresponding Pearson correlation coefficients; red lines, ordinary least-square (OLS) regression; orange lines, LOWESS; and gray lines, orthogonal distance regression (ODR).

*ICOPa* and *ICOPb* knockdown (KD) using RNAi by feeding, individually or combined, was performed to assess the ICOP roles. KD of *ND7*, a gene involved in trichocyst discharge (exocytosis) (Skouri and Cohen 1997) was used as negative control (CTRL). Previously published *ISWI1*-KD data (Singh et al. 2022) were used as a positive control and for comparison. KD efficiency was confirmed using RNA-seq: The target gene expression was substantially reduced compared with the controls in all KDs (Fig. 4A). As the ICOPs are 92% identical at the nucleotide level, we checked for paralog cosilencing. Allowing no mismatches and searching the “*Paramecium tetraurelia* strain 51 transcript (v2.0)” database, ParameciumDB's off-target tool (Arnaiz et al. 2020) predicted a 24 bp window in the *ICOPb* RNAi construct that could cosilence the endogenous *ICOPa* gene (*Paramecium* siRNAs are typically 23 nt, so two possible antisense siRNAs from the *ICOPb* construct could silence endogenous *ICOPa* mRNAs). With the same database and search parameters, the *ICOPa* construct does not have off-target RNAs predicted. We observed cosilencing of the opposing paralogs in the single KDs but to a lesser extent than the target gene (Fig. 4A). We note that the *ICOPb*-KD cultures were asynchronous, in which ∼20% of total cells progressed faster in development and had new MACs, whereas the majority of cells were still in early development. For this, we compared mRNA levels to a similarly asynchronous control KD. *ICOPa*-KD led to 30% lethality, and *ICOPb*-KD led to ∼20% lethality; a double KD of *ICOPa* and *ICOPb* led to ∼65% lethality in the F_1_ generation (Fig. 4B). Additionally, most single KD cells failed to grow at a standard division rate (Fig. 4B, “sick” cells).

PCRs on genomic DNA from the cells that completed MAC development were used to check whether the *ICOP* KDs affect IES excision (Fig. 4C). Longer fragments containing IESs (IES^+^) were amplified in all KD permutations, suggesting ICOPs are essential during genome editing.

Next, we investigated the genome-wide effect of *ICOP* KDs on IES retention using whole-genome sequencing of new MAC-enriched DNA. For each IES a retention score (IRS) was calculated as IES^+^/(IES^+^) + (IES^−^) (IES^+^= reads with IES; IES^−^= reads without IES). Both single and double KDs caused substantially more IES retention than did CTRL-KD. IRS score distributions of *ICOP* KDs were similar to those of *ISWI1*-KD (Fig. 4D) and right-shifted toward higher IRS compared with KDs of *PTIWI01/09*-KD (Fig. 4E, diagonal histograms). In addition, transposon retention was also observed when sequencing reads were mapped against Sardine and Thon transposons (ENA identifier: HE774469) (Supplemental Fig. S5A).

Strong IRS correlation suggests close cooperation between different genome editing molecules. For example, *EZL1* and *PTCAF1*, genes of the *Paramecium* PRC2 complex (Miró-Pina et al. 2022; Wang et al. 2022), have a strong IRS correlation (Swart et al. 2017) when knocked down. Like *ISWI1*-KD, *ICOPa/b*-KD IRSs correlated modestly with other gene KD IRSs (e.g., Fig. 4E). The correlation of *ICOPa/b*-KD was strongest with *ISWI1*-KD (Pearson's correlation = 0.75) (Fig. 4E).

### *ICOPa/b*-KD affects IES excision precision

**Figure 5.**
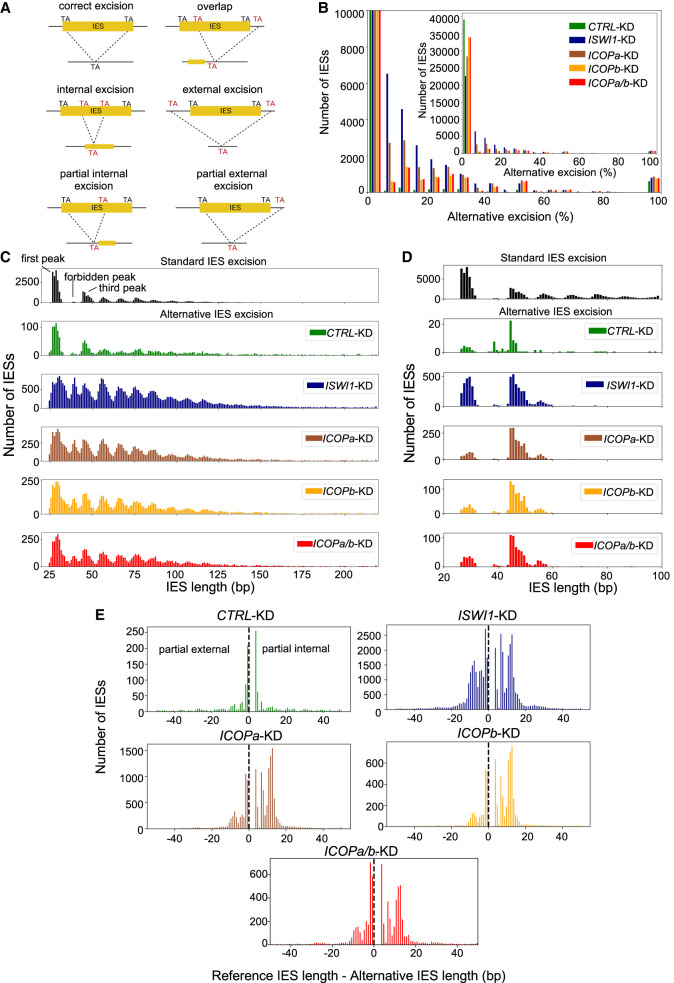
Alternative IES excision in *ICOP* and other relevant KDs. (*A*) Schematic representation of analyzed IES excision events. (*B*) Distribution of genome-wide alternative IES excision (percentage per IES) for different KDs. (*C*) Length distribution of alternatively excised IESs for each KD. The reference length distribution for all IESs is given *above* (“standard IES excision”). (*D*) Origin of alternatively excised IESs in the “forbidden” peak. The reference length is plotted for all alternatively excised 34–44 bp IESs. (*E*) Length distribution of partial external and partial internal alternative excision events for the KDs.

IES excision errors can naturally manifest as alternative excision, occurring at *Paramecium* TA dinucleotides that are not the predominant boundaries (Fig. 5A; Duret et al. 2008). Generally, natural alternative excision levels are low (Fig. 5B,C, CTRL-KD). *ISWI1*-KD substantially enhances alternative excision versus KDs of other genome-editing genes (Singh et al. 2022). Similar to but less than *ISWI1*-KD, *ICOPa*-KD and *ICOPb*-KD increased imprecise excision (Fig. 5; Supplemental Table S2). Previously (Singh et al. 2022), we did not measure alternative excision of IESs for which 100% of the mapped reads were alternatively excised (Supplemental Table S2), thus underestimating alternative excision. Nevertheless, by the old estimation method, the percentage of alternative excision events per IES was highest in *ICOPa-*KD (mean, 7%) and similar between *ICOPb*-KD (mean, 4.2%) and *ICOPa/b*-KD (mean, 4.7%). With the exception of *ISWI1*-KD (mean, 9.2%) (Supplemental Table S2; Singh et al. 2022), this is higher than other KDs (mean, 1.5%–2.4%) (Singh et al. 2022).

The use of alternative TA boundaries changes the excised fragment lengths. The maximum and minimum excised IES lengths were shifted toward the extremes, and alternatively excised IESs were generally longer than the reference length. The alternatively excised IES length distribution resembled the ∼10 bp periodicity characteristic of *Paramecium* IESs, with the striking exception that the “forbidden” peak (Arnaiz et al. 2012) was present in all three *ICOP* KDs, as in *ISWI1*-KD (Fig. 5C). In *ISWI1*-KD, alternative IESs in the “forbidden” peak mainly originated from the first and third peaks, whereas they primarily originated from the third peak in *ICOP* KDs (Fig. 5D). The similarity in alternative excision effects of *ISWI1* and *ICOP* KDs suggests that ISWI1 and ICOP proteins cooperate in precise IES excision.

Furthermore, we examined five possible alternative IES excision events: “partial internal,” “partial external,” “overlap,” “internal,” and “external” (Fig. 5A). Generally, “internal” and “external” are low-frequency events in all KDs (Supplemental Fig. S5B). In the negative control KD, “overlap,” “partial external,” and “partial internal” events were approximately equal at ∼30% each (Supplemental Fig. S5C). This contrasts with *ICOP*s and *ISWI1* KDs, in which “overlap” was infrequent, whereas “partial internal” and “partial external” comprised the largest share of erroneous excision events (Fig. 5E; Supplemental Fig. S5C; Supplemental Table S3). In *ISWI1*-KD, “partial internal” (43%) and “partial external” (42%) events contributed equally, whereas “partial internal” dominated the *ICOP* KDs. The preference was more pronounced in the single KDs (“partial internal”: 57%; “partial external”: 28% for *ICOPa-* and *ICOPb-*KD) than in *ICOPa/b*-KD (“partial internal”: 47%; “partial external”: 34%) (Supplemental Fig. S5C).

### *ICOPa/b*-KD does not alter ISWI1-GFP or GFP-Ptiwi09 localization but affects scnRNAs and iesRNAs

**Figure 6.**
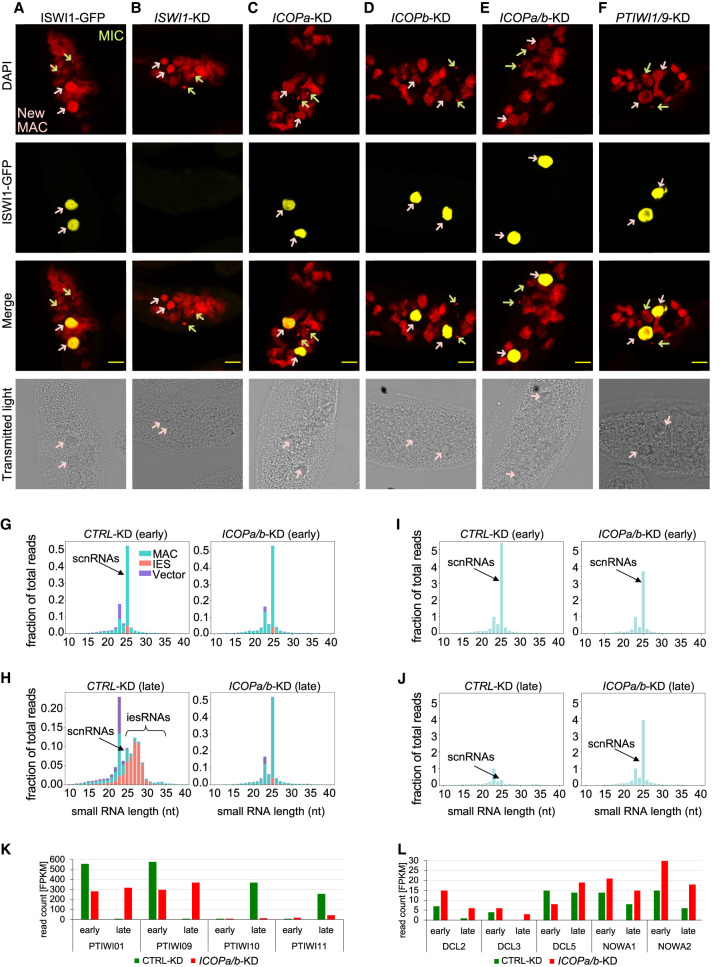
Effects of *ICOPa* and *ICOPb* KDs on ISWI-GFP localization, sRNAs, and gene expression. (*A–F*) Confocal fluorescence microscopy of ISWI1-GFP localization in different gene KD backgrounds. (*A*) Positive control is the ISWI1-GFP-transformed cells without RNAi. Red indicates DAPI; yellow, GFP; green arrow, MIC; and pink arrow, new MAC. Scale bar = 10 µm. (*G*–*J*) sRNA histograms. (*G*,*H*) sRNA reads mapped to the L4440 plasmid sequence (vector), MAC, and IESs. (*I*,*J*) Histogram of MAC genome–matching sRNAs normalized against MAC genome–matching siRNAs. Early indicates 40% of cells have fragmented MAC (T6–T10); late, most cells with visible new MAC (T12–T16). (*K*,*L*) Histogram of mRNA expression levels in FPKM for different development-specific genes.

We knocked down *ICOPa* and/or *ICOPb* to check whether their expression is required for ISWI1-GFP localization. As in control cells with no RNAi (Fig. 6A), ISWI1-GFP localization was not impaired in *ICOP* KDs (Fig. 6C–E). Only in *ISWI1*-KD was the GFP signal entirely lost from the new MAC (Fig. 6B). Conversely, HA-ICOPa and ICOPb-HA localized to the new MACs upon *ISWI1*-KD (Supplemental Fig. S2B) as in non-KD cells. In *Paramecium*, the excision of a subset of IESs is suggested to depend on scnRNAs (Garnier et al. 2004). We tested the dependence of ISWI1-GFP and ICOP-HA localization on scnRNAs by knocking down *PTIWI01/09* (scnRNA Piwis). ISWI1-GFP, HA-ICOPa, and ICOPb-HA localized to the new MAC upon *PTIWI01/09*-KD (Fig. 6F; Supplemental Fig. S2B). This suggests ISWI1 localization is independent of the ICOP proteins and genome scanning.

Conversely, we checked whether *ICOPa/b*-KD influences the small RNA population and, consequently, genome scanning. scnRNAs are generated in MICs well before new MAC development (Lepère et al. 2009). Consequently, scnRNA production should only be affected by the silencing of genes involved in their biogenesis. As expected, in early development (∼40% of cells with fragmented parental MACs), we did not observe a pronounced effect on scnRNA production in *ICOPa/b*-KD compared with the negative control *ND7*-KD (*CTRL*-KD) (Fig. 6G).

KDs of genes like *PTIWI10/11* and *DCL5* directly involved in iesRNA biogenesis inhibit iesRNA production (Sandoval et al. 2014; Furrer et al. 2017). KDs of other genes that inhibit IES excision, like *NOWA1/2*, *PDSG1/2*, *EZL1*-KD, and *PTCAF1* (Arambasic et al. 2014; Ignarski et al. 2014; Lhuillier-Akakpo et al. 2014; Swart et al. 2017) also inhibit iesRNA production, presumably as iesRNAs require excised IESs as substrates for the transcription of their dsRNA precursors (Allen et al. 2017). Here, we also observed inhibition of iesRNA production for *ICOPa/b*-KD (Fig. 6H) in late development.

Comparing the MAC-matching scnRNAs normalized to MAC-matching siRNAs, there was a greater quantity of MAC-matching scnRNAs in the late time point (∼90% of cells with visible new MACs) for *ICOPa/b*-KD than for *CTRL*-KD (Fig. 6I,J). This suggests that MAC-matching scnRNA subtraction, as proposed in the RNA scanning model, was impaired by *ICOPa/b*-KD (Fig. 6J). We also examined sRNA biogenesis-related gene transcription (i.e., *PTIWI*, *DCL*, and *NOWA*) in *ICOPa/b*-KD versus *CTRL*-KD. In late development, *PTIWI10* and *PTIWI11* expression was almost completely lost upon *ICOPa/b*-KD (Fig. 6K), whereas *PTIWI01, PTIWI09*, *DCL2*, *DCL3*, and *NOWA1/2* were upregulated (Fig. 6K,L). Hence, MAC-matching scnRNA enrichment might be caused by scnRNA-associated gene dysregulation.

We also investigated Ptiwi09-GFP localization upon *DCL2/3*-KD, *ISWI1-*KD, and *ICOPa/b*-KD (Supplemental Fig. S6). Without KD, Ptiwi09-GFP localizes first to the parental MAC and cytosol and later to the new MACs during development. Additionally, we observed Ptiwi09-GFP transiently in the swelling MICs before the first meiotic division (Supplemental Fig. S6A). Upon *DCL2/3*-KD, Ptiwi09-GFP failed to enter MICs and the parental MAC, remaining in the cytosol, whereas its localization to the new MACs was unimpaired (Supplemental Fig. S6B). A similar pattern of MIC and old MAC exclusion of Ptiwi09-HA owing to *DCL2/3*-KD was reported previously (Owsian et al. 2022), with much less new MAC localization, but the tag and visualization method differed from ours. Another study reported MIC and old MAC exclusion of Ptiwi09-GFP in *DCL2/3*-KD (Miró-Pina et al. 2023), but with much less cytosolic Ptiwi09-GFP than in our study or in Owsian et al. 2022, possibly owing to lower Ptiwi09-GFP expression. In *ISWI1*-KD and *ICOPa/b*-KD, we observed a tendency for Ptiwi09-GFP to remain longer in parental MAC fragments and an enhanced localization around the MICs during new MAC development compared with non-silenced control cells (Supplemental Fig. S6C,D).

### *ICOPa/b*-KD IES nucleosome density changes are similar to those of *ISWI1*-KD

**Figure 7.**
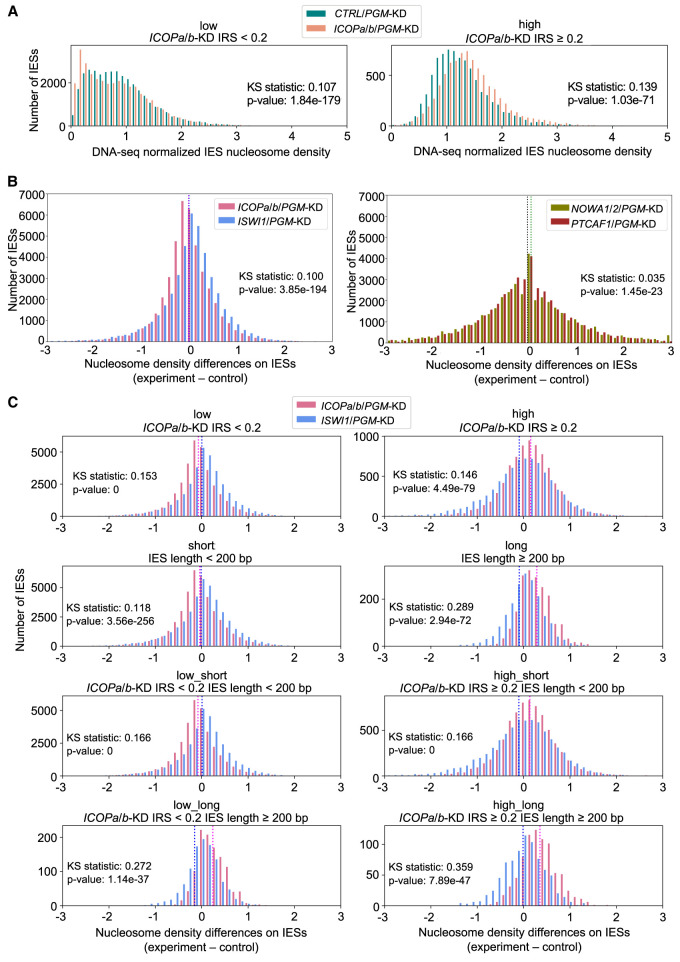
Nucleosome density changes associate with *ICOP* KDs. (*A*) Normalized nucleosome densities across IESs for *ICOPa/b/PGM*-KD and *CTRL/PGM*-KD. IESs are grouped as low (IRS < 0.2) or high (IRS ≥ 0.2) IRS in *ICOPa/b*-KD. (*B*) Nucleosome density differences for all IESs. Means are dashed lines. Magenta indicates *ICOPa/b/PGM*-KD; blue, *ISWI1/PGM*-KD; green, *NOWA1/2/PGM*-KD; and black, *PTCAF1/PGM*-KD. (*C*) Comparison of *ICOPa/b/PGM*-KD and *ISWI1/PGM*-KD in selected IES groups: IESs were grouped by IRS in *ICOPa/b*-KD (low: IRS < 0.2; high: IRS ≥ 0.2) and IES length (short: IES length < 200 bp; long: IES length ≥ 200 bp). IES group is given above the diagrams. Means are dashed lines. Magenta indicates *ICOPa/b/PGM*-KD; blue, *ISWI1/PGM*-KD.

To further investigate the functional contribution of the *ICOP* paralogs to the ISWI1 complex, we analyzed the *ICOP* KD effects on IES nucleosome densities. IESs with high retention in *ICOPa/b*-KD (IRS ≥ 0.2) tended to have higher nucleosome densities (Fig. 7A; Supplemental Fig. S7C,D) in both *ICOPa/b/PGM*-KD and *CTRL/PGM*-KD, similar to our previous observations with other KDs including *ISWI1/PGM*-KD (Singh et al. 2022). The nucleosome density differences (experiment minus control) for *ICOPa/b/PGM*-KD and *ISWI1/PGM*-KD had similar distributions with a narrow peak centered around zero (Fig. 7B; Supplemental Table S4). The comparable distributions for *NOWA1/2/PGM*-KD and *PTCAF1/PGM*-KD were similar to one another but broader and flatter than *ICOPa/b/PGM*-KD (Fig. 7B). This suggests distinct effects of the ISWI1 complex on nucleosome densities and would accord with ICOPa/b and ISWI1 being present in a distinct complex from PTCAF1 and NOWA1/2.

To check effects of IES length and *ICOPa/b*-KD IRS on nucleosome density, IESs were grouped according to these properties. In *ICOPa/b/PGM*-KD and *ISWI1/PGM*-KD, nucleosome density differences were most prominent for long and/or ICOPa/b-dependent IESs (Fig. 7C). In the *ISWI1/PGM*-KD, there was no clear trend toward higher or lower nucleosome densities, whereas in *ICOPa/b/PGM*-KD, there tended to be higher nucleosome densities in the experimental sample (Fig. 7C; Supplemental Table S4). This shift toward higher nucleosome densities was also observed for *PTCAF1/PGM*-KD (Supplemental Fig. S8; Supplemental Table S4), indicating this effect is not specific to components of the ISWI1 complex.

## Discussion

In this study, we identified and analyzed the role of the ICOPa and ICOPb subunits that, together with the ISWI1 protein subunit, appear to form a developmental genome editing complex in *Paramecium*. ICOPa and ICOPb are highly divergent from other proteins, lacking homology or additional domains detectable by routine search methods. In such cases, it may be helpful to use software like HHpred, using pairwise HMM comparisons for distant homology searches (Zimmermann et al. 2018). Thus, we identified a highly divergent WSD motif in ICOPs (Fig. 1). The WSD motif is found in proteins that are subunits of the ISWI complex in several organisms (Fig. 1D; Toto et al. 2014).

We provided evidence using *Paramecium* and *E. coli* that ISWI1 forms a complex with the ICOP proteins (Fig. 2). The observations of proteins overexpressed in *E. coli*, lacking *Paramecium* proteins, support direct ISWI1–ICOP binding. ISWI1 coimmunoprecipitates with both paralogs. ICOPb was not enriched in HA-ICOPa co-IP, whereas ICOPb-HA co-IP has low ICOPa enrichment (Supplemental Fig. S3D). Thus, despite their ability to interact directly (Fig. 2D), it is likely that ISWI1 might typically form complexes with either ICOP subunit.

Although WSD's GxD aspartate is proposed to determine ISWI-WSD motif–containing protein interaction (Aravind and Iyer 2012), to our knowledge, no supporting experimental evidence exists for this. In an Ioc3 crystal structure, a WSD motif–containing protein of yeast, the GIQ lacks the third acidic residue and forms no polar interactions with ISW1a (Fig. 3I). Our heterologous expression studies show that GxD signature mutation or deletion does not completely abolish ICOP–ISWI interaction (Fig. 3C,D). Furthermore, AlphaFold2 modeling predicted the interaction of the ICOPs at the ISWI1 N terminus, mediated by a helix–turn–helix motif rather than the GxD (Fig. 3F,G). Better structural prediction software and experimental approaches will be needed to determine precisely how the proteins interact in this complex.

Along with strong iesRNA production inhibition, *PTIWI10*/*11* expression was abolished by the *ICOP* KDs. As these genes are transcribed in the developing MAC (Furrer et al. 2017), the loss of *PTIWI10/11* expression could either be because of IES retention in their promoters or because of nonsense-mediated decay (NMD) of mRNA triggered by IES retention in the CDS (Furrer et al. 2017; https://www.ebi.ac.uk/ena/browser/view/SRX309864?show=reads [accessed August 30, 2022]; Bazin-Gélis et al. 2023) sRNA sequencing also revealed that the MAC-specific scnRNAs are elevated in *ICOPa/b-*KD compared with the control (Fig. 6H,J). The same phenomenon has been observed in *NOWA1/2*-KD (Swart et al. 2017) and *PTCAF1*-KD (Ignarski et al. 2014). NOWA1/2 is involved in genome scanning (Nowacki et al. 2005), whereas PTCAF1 is a part of the PRC2 complex employed in H3K27me3 deposition during IES excision (Ignarski et al. 2014; Miró-Pina et al. 2022; Wang et al. 2022). Previous studies suggest that elevated MAC-specific scnRNA levels are caused by PTCAF1 inhibition in the old MAC during scanning (Ignarski et al. 2014).

With the caveat of the lack of replicates, unlike *PTIWI10/11* (iesRNA Piwis), genes associated with scnRNAs, notably *PTIWI01*/*09*, were modestly upregulated in the late developmental stage upon *ICOPa/b*-KD, potentially by inhibiting MAC genome–matching scnRNAs from degradation. Although we observed subtle differences in Ptiwi09-GFP localization after *ICOPa/b*-KD, this would have to be replicated and the possible mechanism investigated in the future. Likewise, the reasons for Ptiwi09 localization changes owing to *DCL2/3*-KD that we and others (Owsian et al. 2022; Miró-Pina et al. 2023) have observed also require more detailed investigation. Furthermore, it would also be worth revisiting the RNA scanning model in *Paramecium*, rigorously examining key details not yet directly established, for example, what substrates the scnRNAs pair with.

It would also be worth investigating the expression of *PTIWI01*/*09* and related genome editing genes (e.g., *NOWA1/2* and *PTCAF1*) for other KDs to compare to those in *ICOPa/b*-KD. However, it is clear that the IES retention in *ICOPa/b*-KD is substantially stronger than in *PTIWI* KDs (Fig. 4E) and also exhibits enhanced alternative excision (Fig. 5). Thus, altered expression of the *PTIWIs* and other genome editing genes cannot account for most of the observed *ICOPa/b*-KD effects, irrespective of whether the development-specific sRNA levels or their MAC:IES ratios are altered.

Most IESs are likely transposon remnants (Sellis et al. 2021; Seah et al. 2023) that decayed beyond recognition owing to their efficient developmental excision (Sellis et al. 2021). One-third of all IESs are 26–28 bp in length and are proposed to be short enough to allow direct interaction of two PGMs without DNA looping (Arnaiz et al. 2012). Longer IESs require DNA looping, causing 34–44 bp IESs in the “forbidden” peak to be highly underrepresented, either too long for the direct PGM subunits’ interaction without looping or too short for DNA looping to permit this interaction. Similar to ISWI1, *ICOP*-KDs caused both IES retention and elevated alternative IES excision (Figs. 4, 5).

Generally, in genome editing gene KDs, alternative excision levels do not exceed the background (Singh et al. 2022), but are enhanced by ISWI1 KD. This led to the emergence of “forbidden” peak length IESs. In the *ICOP* KDs, the alternatively excised IESs in the “forbidden” peak mainly originated from the subsequent peak. This aligns with the observation that partial internal excision, leading to shorter lengths, dominated alternative excision events in *ICOP* KDs (mainly in the single KDs). In *ISWI1*-KD, partial internal and external excision contributed equally to the alternatively excised IESs and the “forbidden” peak. The difference in excision preference might be caused by ISWI's ability to move nucleosomes on its own (Havas et al. 2000; Längst and Becker 2001). Some nucleosome repositioning may still happen via ISWI1 in the *ICOP* KDs, although not as effectively as with the ICOPs. However, in *ISWI1*-KD, in which nucleosome repositioning fails, IES removal occurs at the next available TA, whether internal or external.

We observed that nucleosome density difference distributions for *ICOPa/b/PGM*-KD and *ISWI1/PGM*-KD were sharply peaked, indicating generally little difference in nucleosome density on IESs irrespective of the ISWI1 complex presence (Fig. 7B). However, *NOWA1/2/PGM*-KD and *PTCAF1/PGM*-KDs showed broader distributions, implying that IES nucleosome densities are less influenced by the ISWI1 complex components’ downregulation than by the downregulation of other genes. Because nucleosome densities do not capture exact nucleosomal positions, nucleosome positions rather than the number of nucleosomes may change in *ICOPa/b/PGM*-KD and *ISWI1/PGM*-KD. However, this cannot be properly investigated by current computational methods owing to the inability to distinguish between most old and new MAC sequences, as well read alignment accuracy limitations at IES boundaries.

*NOWA1/2/PGM*-KD and *PTCAF1/PGM*-KDs might have stronger effects on nucleosome density differences because *NOWA1* and *PTCAF1* are expressed earlier than the ISWI1 complex and localize to both maternal and developing MACs (Nowacki et al. 2005; Ignarski et al. 2014). Therefore, observed nucleosome density differences could either be because disruption of events downstream from NOWA1 and PTCAF1 functions or because of inter-generational nuclear cross talk effects on gene regulation as proposed recently (Bazin-Gélis et al. 2023). Irrespective, a clear difference on both chromatin and IES excision can be observed between the ISWI1 complex and other genome editing components, indicating a distinct role for ICOPs and ISWI1 on nucleosomes.

ICOP paralogs might contribute to the directionality of the remodeling complex, as shown for *Drosophila* Acf1, a protein that regulates ISWI-containing complex CHRAC directionality (Eberharter et al. 2001). In contrast to ISWI1, ICOP KDs caused a preference both for partial internal excision (Supplemental Fig. S5C) and for higher nucleosome densities on long/highly retained IESs (Fig. 7C). Higher nucleosome densities might be a direct cause for preferred partial internal excision.

We previously proposed a “clothed” model for IES excision, in which mispositioned nucleosomes change the accessibility of the IES boundaries to the PGM excision complex (Singh et al. 2022). Assuming that the cooperating PGMs cannot interact across a nucleosome without a sufficiently long DNA loop, partial internal excision might be preferred if a nucleosome is located on a TA boundary because an alternative TA lying within the IES might be more easily accessible than one outside.

Besides nucleosome positioning, precise IES boundary targeting might also depend on the DNA topology, which influences protein binding and can be exploited in regulation (Baranello et al. 2012). Some ISWI family chromatin remodelers can change the DNA topology (Havas et al. 2000), which might cause the PGM complex to recognize the wrong TA dinucleotides if alterations in chromatin remodeling occur. This would also explain how the “forbidden” peak can emerge. According to the original “naked” DNA model, the PGM excision machinery struggles to excise 34–44 bp fragments (Arnaiz et al. 2012). However, if the DNA helix conformation changes, the PGM complex working distance might correspond to the forbidden length. It seems that the ICOPs can partially compensate for each other because their double KD resembled the *ISWI1*-KD more than their single KDs in terms of cell survival (Supplemental Fig. S3A) and IES retention and alternative excision effects (Figs. 4D, 5B; Supplemental Fig. S5C). We thus propose that the ICOP proteins assist ISWI1's function in precise genome editing, either by nucleosome sliding or by DNA topology changes.

In *Paramecium*, linker DNA between somatic nucleosomes was shown to be extremely short at just a few base pairs (Gnan et al. 2022), and no linker histone H1 was detected (Drews et al. 2022b). Furthermore, histone modifications characteristic of euchromatin and heterochromatin in other eukaryotes did not show the expected relations with active and repressive gene expression (Drews et al. 2022b). *Paramecium* MIC and MAC nucleosome properties, like their distribution and dynamics, still need more thorough investigation. Future studies enabling more precise nucleosome positioning, potentially via isolation from flow-sorted MACs, will be essential to determine how nucleosome occupancy and movements by complexes like ISWI1–ICOP affect the targeting of natural genome editing.

## Methods

### Culture cultivation and RNAi assays

Culture cultivation and RNAi assays are described in Supplemental Methods.

### DNA microinjection and localization

The standard DNA microinjection protocol was followed (Beisson et al. 2010). Because ICOPa and ICOPb fusion gene expression with endogenous flanking regulatory regions failed, those of ISWI1 (Singh et al. 2022) were used instead. Human influenza hemagglutinin (HA) was fused N-terminally to ICOPa and C-terminally to ICOPb. ISWI1-GFP plasmid is described by Singh et al. (2022). The GFP-PTIWI09 plasmid was a gift from the Nowacki laboratory. Cells were collected during different stages of autogamy and either stored in 70% ethanol at −20°C or directly fixed with 2% formaldehyde (PFA) in PHEM (PIPES, HEPES, EGTA, magnesium sulfate), washed (2 × 5 min at room temperature [RT]). Five percent BSA with 0.1% Triton X-100 in Tris-buffered saline with 10 mM EGTA and 2 mM MgCl_2_ (TBSTEM) was used for blocking (1 h, RT). Cells were stained overnight at 4°C with a primary anti-HA antibody (Santa Cruz sc-7392) followed by washing and secondary antimouse Alexa-594 (Biozol BLD-405326) or antimouse Alexa-568 (Thermo Fisher Scientific A11004) incubation (1 h, RT). After washing, cells were counterstained with 4,6-diamidino-2-2-phenylindole (DAPI) in 5% BSA with 0.1% Triton X-100-TBSTEM. Cells were mounted with 40 µL of ProLong gold antifade mounting medium (Invitrogen). Images were acquired with a Leica SP8 confocal microscope system with a 60× oil objective (NA 1.4). Images were analyzed using Fiji (version 2.9.0/1.53t) (Schindelin et al. 2012). Macros used for image analysis are available at GitHub (https://github.com/Swart-lab/ICOP_code/tree/main/Postprocessing_IF) and as Supplemental Code.

### co-IP and western blotting

co-IPs and western blots were done as previously described (Singh et al. 2022) using late-stage lysates. Sonication used an MS72 tip on a Bandelin Sonopulse device with 52% amplitude for 15 sec. For non-cross-linked samples, cells were lysed using sonication on ice after washing with 10 mM Tris (pH 7.4) in a resuspension of 2 mL lysis buffer. Pulldown fractions were resolved on 12% SDS-PAGE gels. One percent of total lysates were loaded as input, optionally 1% of supernatant after beads incubation as unbound, and 30% (Fig. 2) or 20% (Supplemental Fig. S3) of the total IP samples was loaded.

An anti-HA antibody (1:500, Santa Cruz sc-7392 HRP) and anti-GFP antibody (1:2000, Abcam ab290) incubation was done overnight at 4°C. The secondary antibody, goat-antirabbit HRP conjugated (Merck Millipore 12-348), was incubated for 1 h at RT. Membranes were screened using AI600 (GE Healthcare) after incubation with an HRP substrate (Millipore 42029053) for 2–5 min.

### Protein expression in *E*. *coli*

Plasmids used for *E. coli* expression are detailed in Supplemental Methods. Fifty milliliters of ZY medium (Studier 2014) containing appropriate antibiotics was inoculated with 100 µL of transformed *E. coli* culture. Cultures were grown at 37°C at 180 rpm until an OD600 of two was reached. Afterward, the temperature was decreased to 20°C for overnight protein expression. Two milliters of culture was centrifuged at 4000*g* at 4°C, and pellets were frozen at −80°C.

### Recombinant protein coprecipitation

Cell pellets were resuspended in 1 mL of lysis buffer: 20 mM Tris (pH 7.5), 100 mM NaCl for GST pulldown or 20 mM Tris (pH 7.5), 100 mM NaCl, 20 mM imidazole, 1 mM DTT for His pulldown. Twenty percent amplitude (0.5 sec on, 0.5 sec off) with an MS72 tip (Bandelin Sonopulse) was used for sonication, followed by centrifugation (21130*g*, 15 min, 4°C) to recover the supernatant for pulldown. Thirty microliters of beads (Serva 42172.01/42318.01) were washed, equilibrated with lysis buffer, loaded with protein supernatant, and incubated for 1 h or overnight at 4°C using gentle shaking. After three washes in lysis buffer, the enriched protein was eluted from beads by adding 30 µL of 2× protein loading buffer (100 mM Tris-HCl at pH 6.8, 4% [w/v] SDS, 20% glycerol, 0.2 M DTT) and boiling for 10 min. The supernatant was loaded on a 10%–12% SDS-PAGE gel. One percent of the total lysate was loaded as input, and 20% of the total pulldown was loaded in the IP fraction; 1:4000 rabbit anti-GST antibody (Sigma-Aldrich G7781) and mouse anti-His (1:2500, BioLegend 362601) were diluted in 5% BSA in 1× PBS + 0.2% Tween 20 for blotting; and 1:5000 reciprocal secondary antibody incubation was done for 1 h at RT.

### DNA and total RNA extraction and sequencing

Standard methods were used to isolate macronuclear DNA and total RNA for sequencing (for details, see Supplemental Methods).

### IES retention and alternative boundary analysis

IES retention scores and alternative excision were calculated as previously described (for details, see Supplemental Methods; Singh et al. 2022).

### Nucleosome density analysis

For nucleosomal DNA isolation and sequencing procedures, see the Supplemental Methods. Nucleosome densities were calculated as previously described (Singh et al. 2022), using a double KD with the focal gene (e.g., ICOPa or ND7) and PGM, as this is necessary to retain sufficient IES sequences in order to map both nucleosomal and regular DNA-seq reads to them. As previously, we focused on IES-mapping reads because the old and new MAC sources of MAC-mapping reads are indistinguishable. Because of the experimental nuclear/nucleosome isolation procedure, most IES-mapping reads should be from the developing new MAC rather than the MIC. For further details see the Supplemental Methods.

### sRNA analysis

sRNA-seq processing and analysis are described in the Supplemental Methods.

### Structure prediction with AlphaFold

Protein structures were predicted with AlphaFold multimer version 2.2.0 and 2.3.0 (Evans et al. 2021; Jumper et al. 2021). Protein sequences provided as input are listed in Supplemental Table S8. All predictions were computed on the high-performance computer “Raven,” operated by the Max-Planck Computing and Data Facility in Garching, Munich, Germany. PDB files are available from Edmond, the Max Planck Open Research Data repository, SourceData_Fig3 (Singh 2023).

## Data access

All whole-genome-sequencing data, small RNA sequencing data, and mRNA sequencing data have been submitted to the European Nucleotide Archive (ENA; https://www.ebi.ac.uk/ena/browser/home) under accession number PRJEB64685. All raw protein MS data have been submitted to the ProteomeXchange Consortium (https://www.proteomexchange.org/) via the PRIDE partner repository (https://www.ebi.ac.uk/pride/) under the project identifier PXD046704. All original images corresponding to gels and microscopy and other data can be accessed from the Max Planck Open Research Data Repository, Edmond (https://doi.org/10.17617/3.ZBOLU8).

## Supplemental Material

Supplement 1

Supplement 2

Supplement 3
