# Supplementary material for: ISWI1 complex proteins facilitate developmental genome editing in Paramecium
Source: Genome Res. 2025 Jan;35(1):93–108. doi: 10.1101/gr.278402.123 (PMC11789628; doi:10.1101/gr.278402.123)
Supplement: Supplement 1 [file Supplemental_Figures_S1_S8.pdf]

Supplemental Figure S1

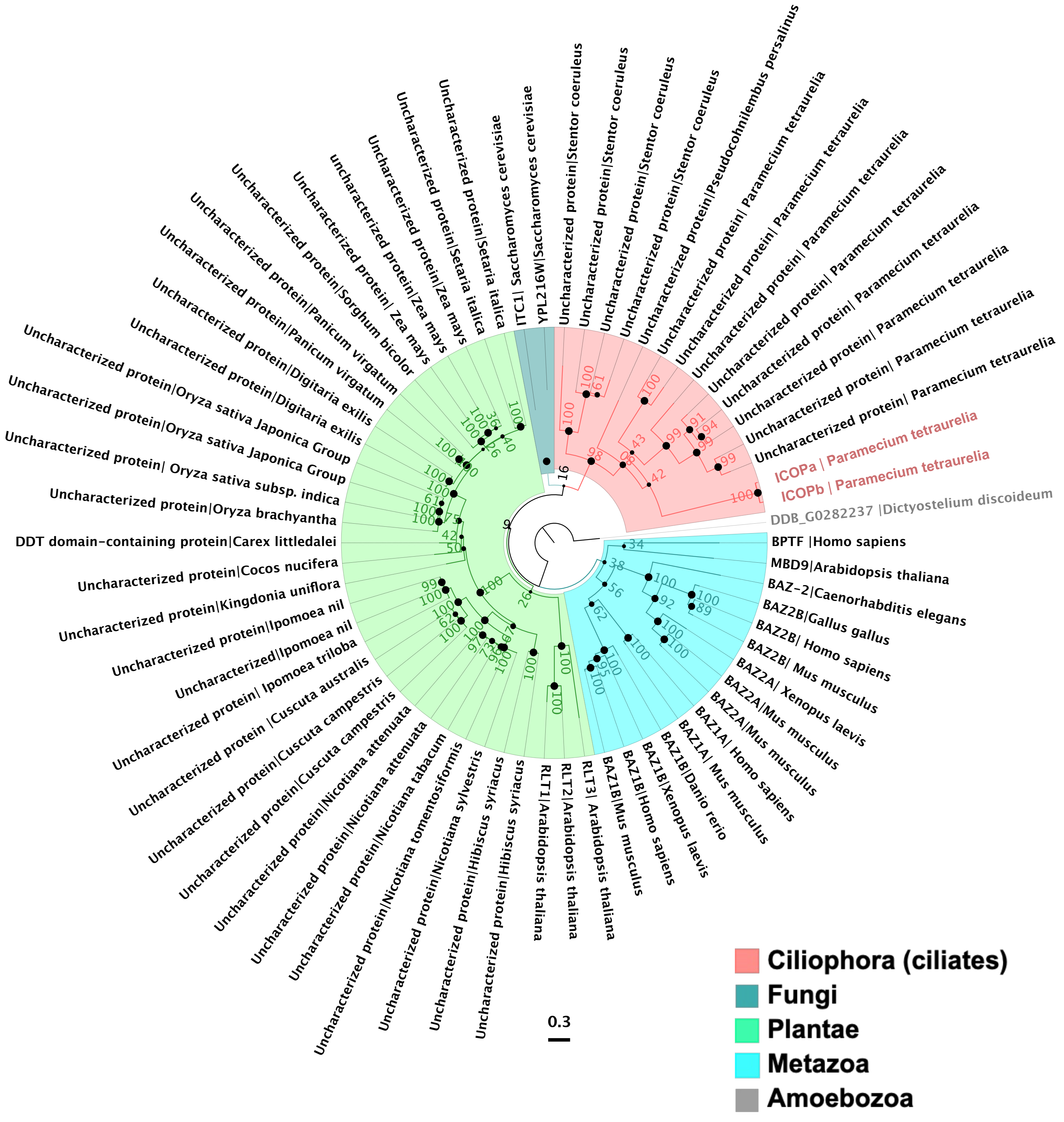

Supplemental Figure S1: WSD motif-containing proteins are highly diverse.

Phylogenetic analysis of proteins with WSD motif in selected organisms. Node bootstrap values are labeled, and the '•' size corresponds to node values. The tree is rooted at the *Dictyostelium discoideum* sequence (gray). Scale bar is 0.3 amino acid substitutions per site. ICOPa and ICOPb (salmon).

Supplemental Figure S2

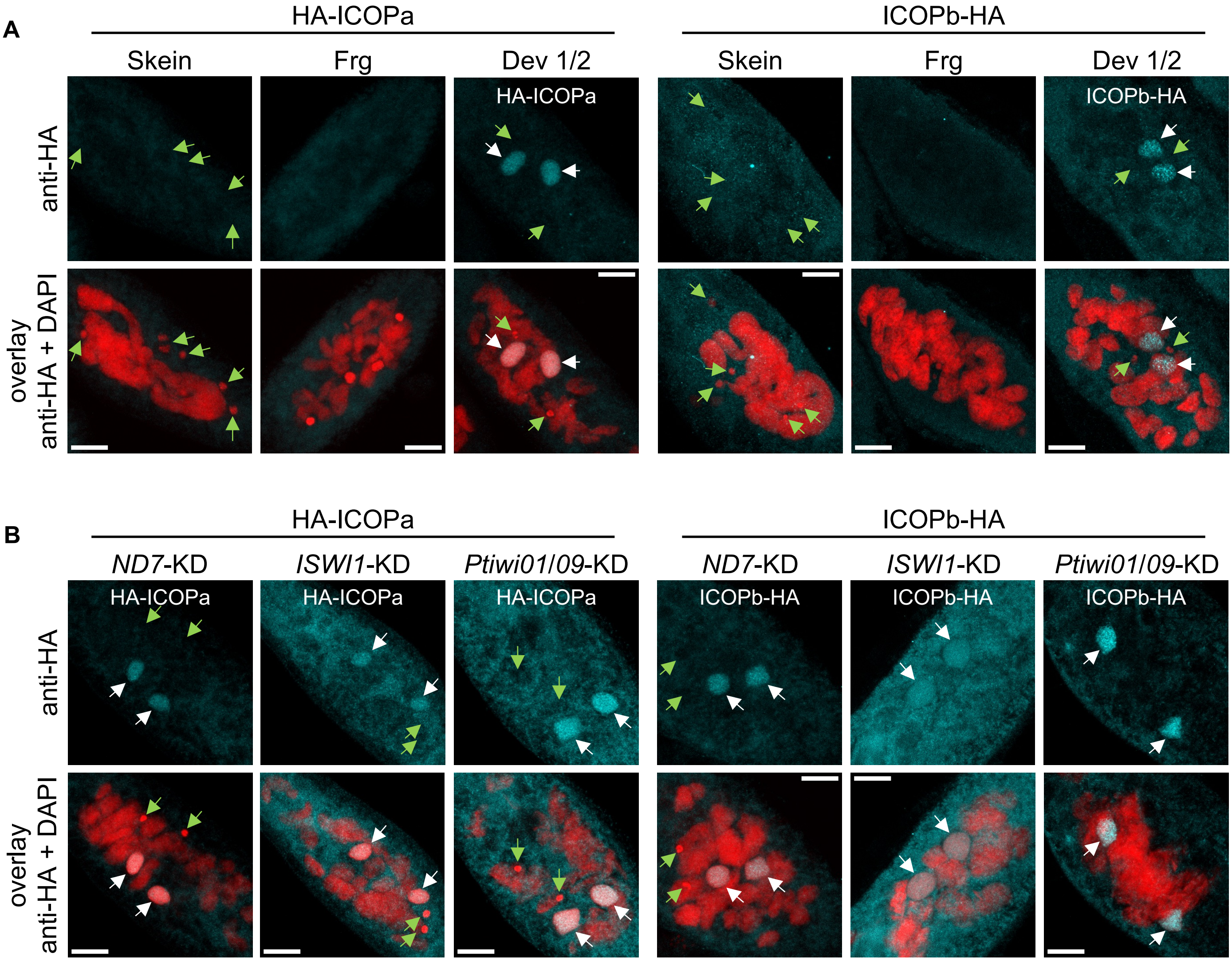

Supplemental Figure S2: ICOP localization.

(A) & (B) Confocal fluorescence microscopy images of HA-ICOPa and ICOPb-HA localization at different developmental stages (A) or upon KDs (B). Maximum intensity projections of z-planes. Red: DAPI, Cyan: HA. Green arrow: MIC. White arrow: new MAC. Brightness and contrast in HA-channel are constant across all HA-ICOPa and across all ICOPb-HA images. Scale bar = 10 µm.

Supplemental Figure S3

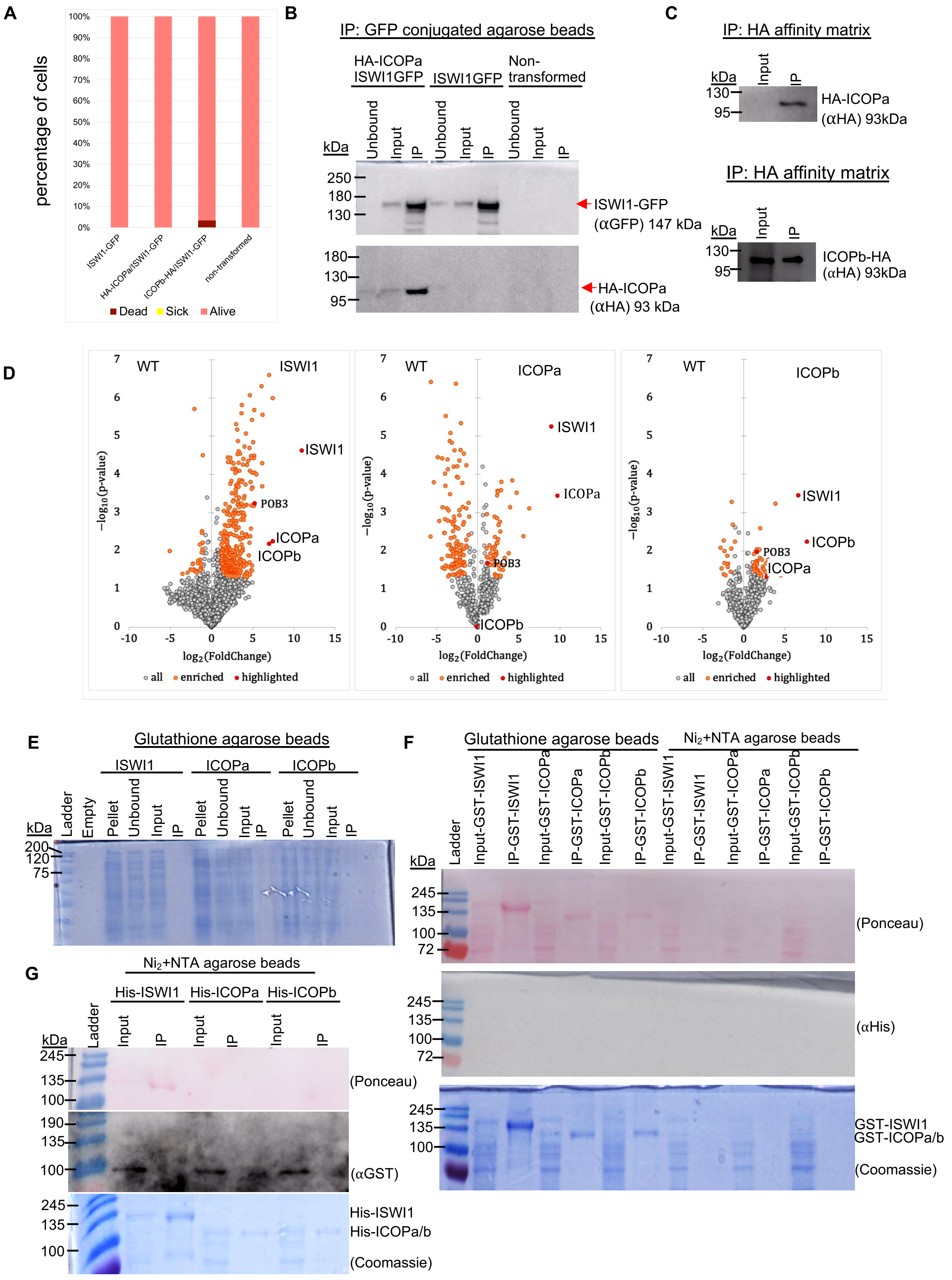

Supplemental Figure S3: ICOP paralogs interact with ISWI1.

(A) Survival assay of F1 generation of the ISWI1-GFP, HA-ICOPa/ISWI1-GFP ,and ICOPb-HA/ISWI1-GFP transformed cell lines, non-transformed cell lines are used as control. Alive (pink): normal division rate (4 divisions/day). Sick (red): slower division rate (<3 divisions/day). Dead (cayenne): no cells. (B) Western blot on co-IP of HA-ICOPa/ISWI1-GFP co-transformed, ISWI1-GFP transformed and non-transformed, wild-type *Paramecium*. (C) Western blot of co-IP of HA-ICOPa and ICOPb-HA overexpressed in paramecia. (D) Volcano plots showing protein enrichment of mass spectrometry (MS) analysis for ISWI1-GFP (left), HA-ICOPa (middle), and ICOPb-HA (right) co-IP. (E) to (F): Pulldowns on overexpressed recombinant proteins in *E. coli*. (E) Coomassie staining of untagged ISWI1, ICOPa and ICOPb. (F) Western blot and Coomassie staining of GST-tagged recombinant protein pulldowns; Ponceau-stained membranes probed with anti-His antibody. (G) Western blot and Coomassie staining of His-tagged recombinant proteins; Ponceau-stained membranes probed with anti-GST antibody.

Supplemental Figure S4

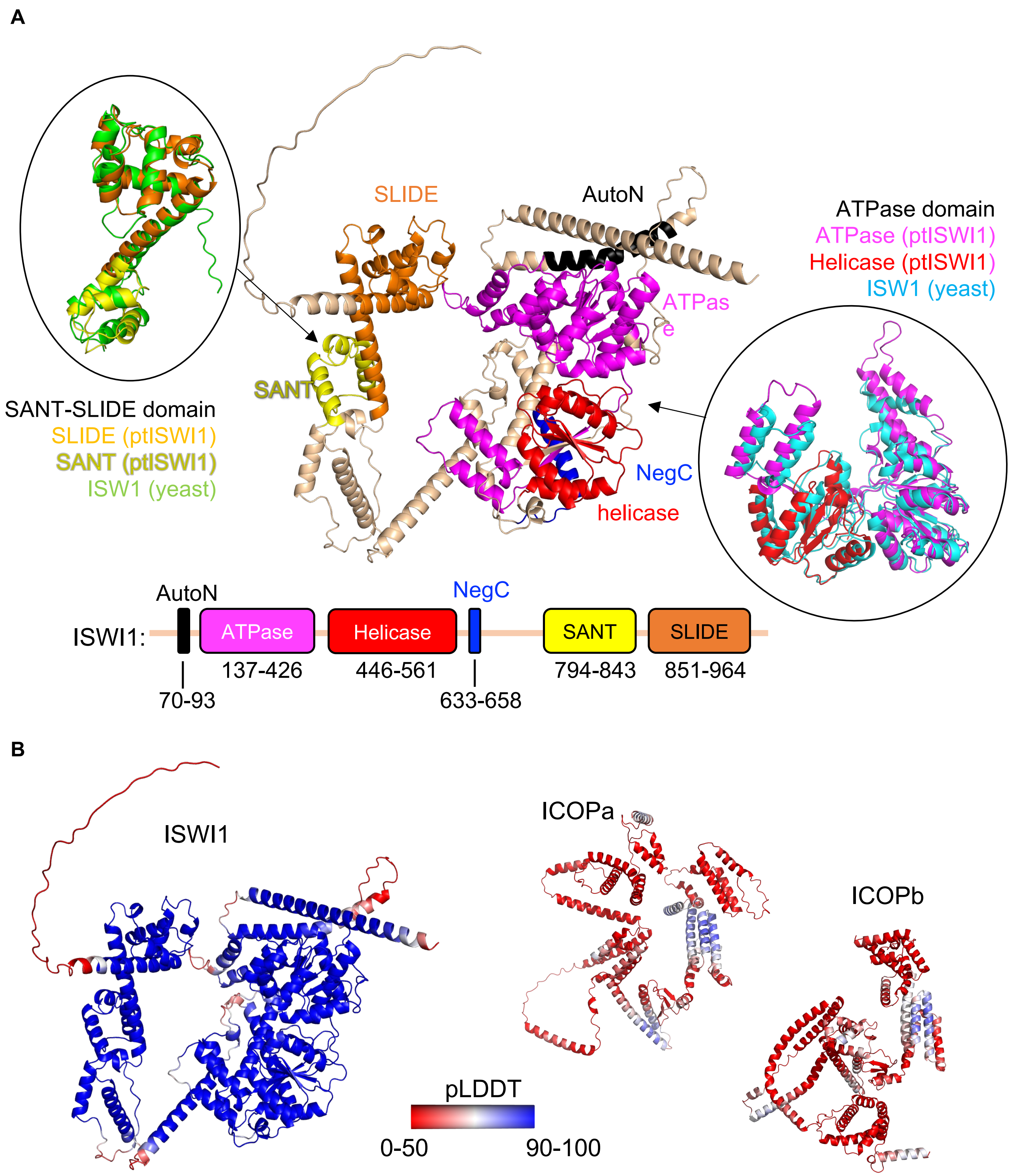

Supplemental Figure S4: ISWI1 and ICOP structure predictions.

(A) and (B) AlphaFold (version 2.2.0) structure predictions. (A) Domains in *Paramecium* ISWI1. ATPase and Helicase are superimposed with a published structure of N-terminal ISWI from yeast (PDB accession number 6JYL) (color: cyan) and SANT-SLIDE domains are superimposed with ISW1a (delATPase) from yeast (PDB accession number 2Y9Y) (color: green). (B) Structure prediction confidence for ISWI1, ICOPa, and ICOPb. Models are colored by predicted local distance difference test (pLDDT). pLDDT ≤ 50: red. pLDDT ≥ 90: blue.

Supplemental Figure S5

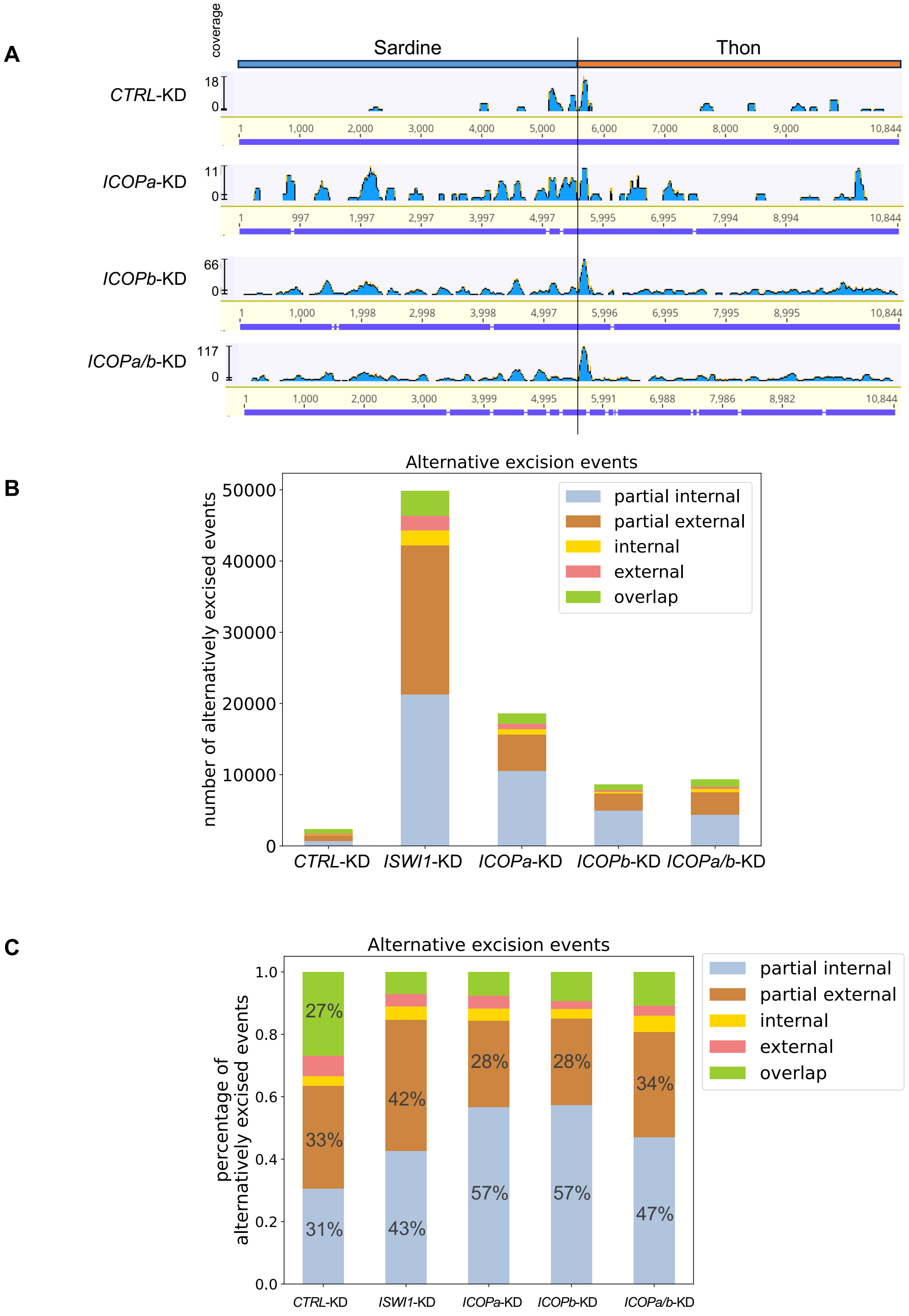

Supplemental Figure S5: DNA elimination events.

(A) Geneious screenshot of DNA-seq reads mapped to Sardine and Thon transposons (ENA HE774469) upon different knockdowns.

(B & C) Stacked bar graphs of alternative excision events in *ISWI1*-KD, *ICOPa*-KD, *ICOPb*-KD and *ICOPa/b*-KD. *ND7*-KD was used as a control (*CTRL*-KD). (B) Absolute and (C) relative abundance of alternative excision events occurring upon KDs.

Supplemental Figure S6

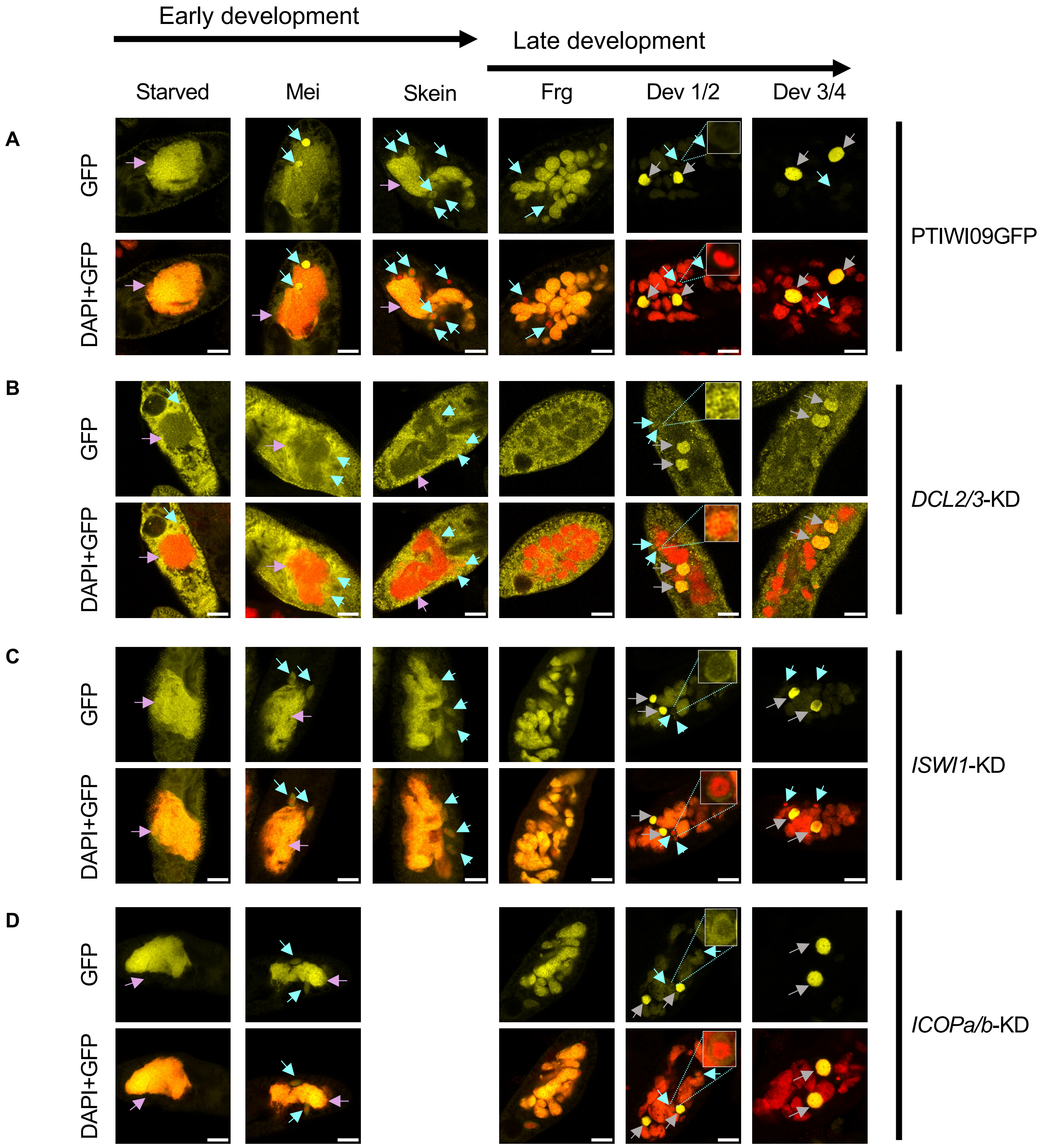

Supplemental Figure S6: Confocal microscopy images of PTIWI09GFP localization upon different knockdowns.

PTIWI09GFP localization without any knockdown (A), upon *DCL2/3*-KD (B), upon *ISWI1*-KD (C), upon *ICOPa/b*-KD (D). MIC is cropped, magnified, and contrast adjusted in Dev1/2 panel for better visualization. Red: DAPI. Yellow: GFP. Purple arrow: Maternal MAC. Cyan arrow: MICs. Grey Arrow: Developing MACs. All channels were optimized for the best visual representation. Scale bar = 10 μm. Starved: induction of autogamy (T0), Mei: MIC meiosis (T3-T5), Skein: beginning of macronuclear fragmentation (T4-T6), Frg: fragmentation of maternal MAC (T6-T10), Dev1/2: visible new MAC (T10-T12), Dev3/4: larger new MAC (T12-T16)

Supplemental Figure S7

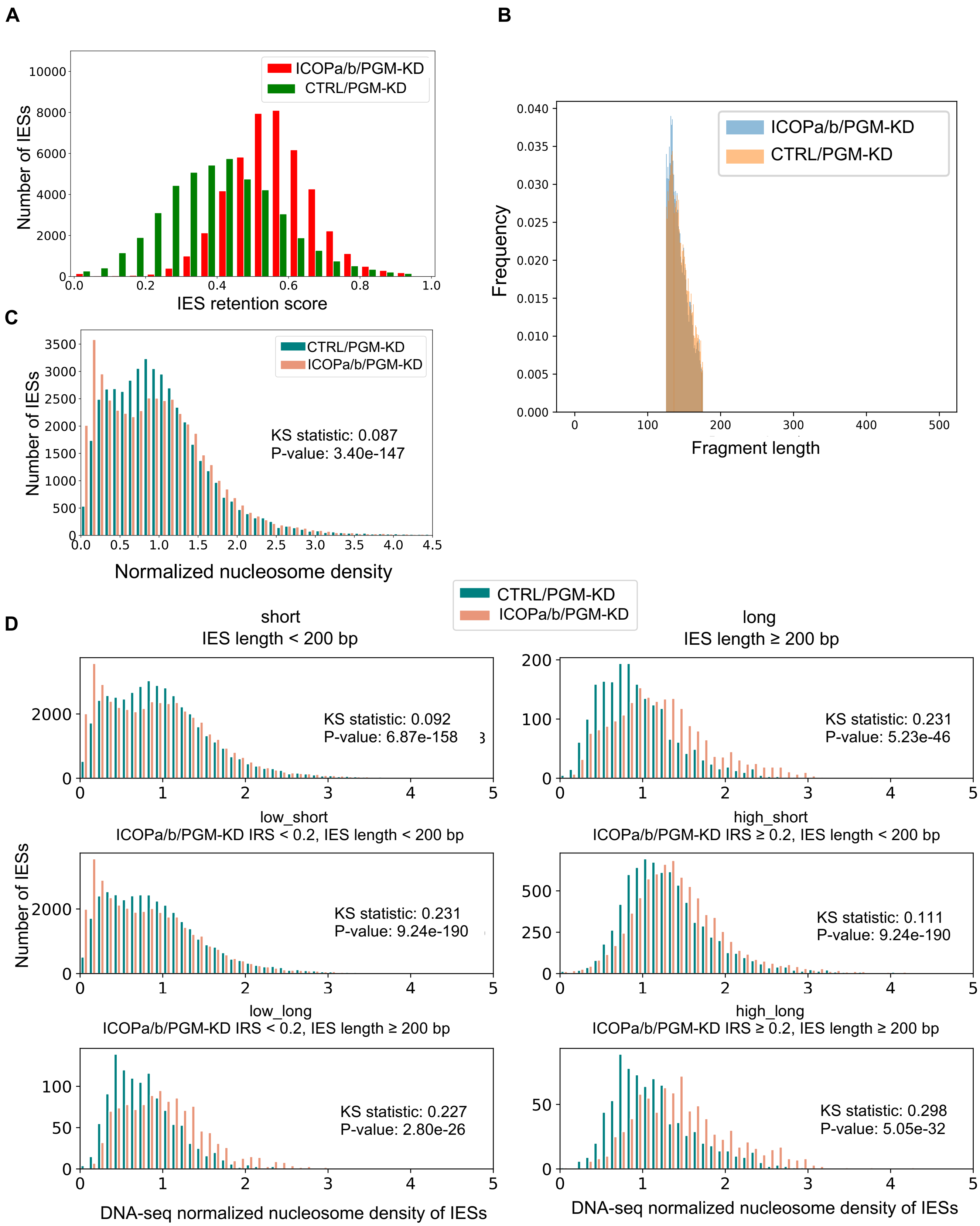

Supplemental Figure S7: Nucleosome densities for *ICOPa/b/PGM*-KD and *CTRL/PGM*-KD.

(A) IRS histogram for *ICOPa/b/PGM*-KD and *CTRL/PGM*-KD. (B) Size distribution of reads mapped to scaffold51_9 for *ICOPa/b/PGM*-KD and *CTRL/PGM*-KD. (C) Nucleosome densities of all IESs in *ICOPa/b/PGM*-KD and *CTRL/PGM*-KD. (D) Nucleosome densities of selected IES groups in *ICOPa/b/PGM*-KD and *CTRL/PGM*-KD. IESs were grouped by IES retention score (IRS) in *ICOPa/b*-KD (low: IRS < 0.2; high: IRS ≥ 0.2) and IES length (short: IES length < 200 bp; long: IES length ≥ 200 bp). IES group is given above the diagrams.

Supplemental Figure S8

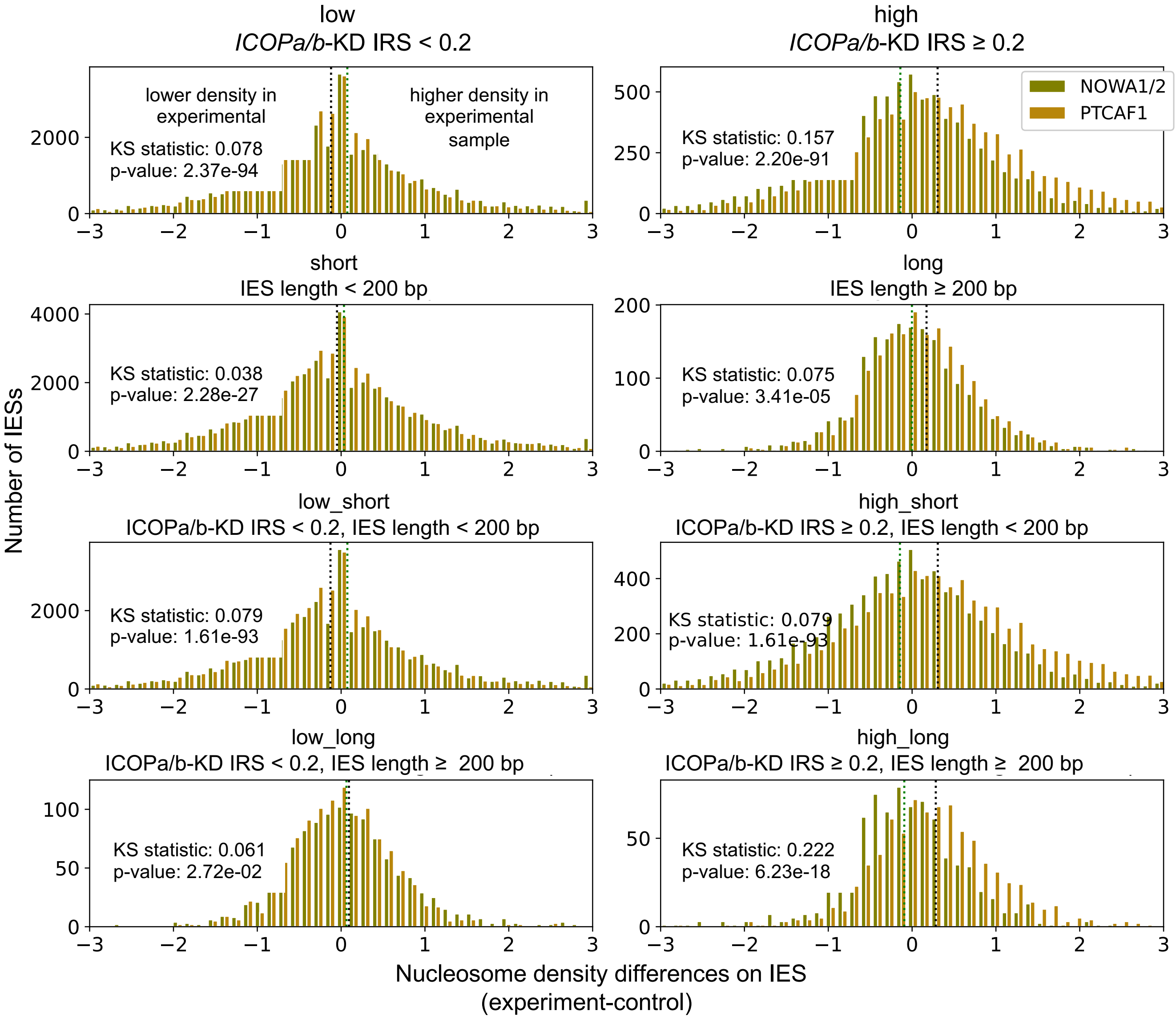

Supplemental Figure S8: Nucleosome density differences for *NOWA1/2/PGM*-KD and *PTCAF1/PGM*-KD.

Comparison of *NOWA1/2/PGM*-KD and *PTCAF1/PGM*-KD nucleosome density differences in selected IES groups: IESs were grouped by IES retention score (IRS) in *ICOPa/b*-KD (low: IRS < 0.2; high: IRS ≥ 0.2) and IES length (short: IES length < 200 bp; long: IES length ≥ 200 bp). The specification for each IES group is given above the individual diagrams. Means are indicated as dashed lines (*NOWA1/2/PGM*-KD: green; *PTCAF1/PGM*-KD: black).
