# Supplementary material for: ISWI1 complex proteins facilitate developmental genome editing in Paramecium
Source: Genome Res. 2025 Jan;35(1):93–108. doi: 10.1101/gr.278402.123 (PMC11789628; doi:10.1101/gr.278402.123)
Supplement: Supplement 2 [file Supplemental_Methods_III.docx]

ISWI1 complex proteins facilitate developmental genome editing in *Paramecium*

Aditi Singh^1,*,#^, Lilia Häußermann^1,*^, Christiane Emmerich^1^, Emily Nischwitz^2^, Brandon KB Seah^1^, Falk Butter^2,3^, Mariusz Nowacki^4^, Estienne C. Swart^1,#^

Table of contents

[**Supplemental Methods 1**](#_heading=h.3znysh7)

[Culture Cultivation 1](#_heading=h.2et92p0)

[RNAi assays 1](#_heading=h.tyjcwt)

[HHpred identification of protein domains 2](#_heading=h.3dy6vkm)

[Phylogenetic analysis of ICOPa and ICOPb 3](#_heading=h.1t3h5sf)

[Mass spectrometry analysis 3](#_heading=h.4d34og8)

[Plasmids and vectors for recombinant protein expression assay 5](#_heading=h.2s8eyo1)

[Total RNA extraction and mRNA sequencing. 5](#_heading=h.17dp8vu)

[Knockdown efficiency validation using RNA-seq 6](#_heading=h.3rdcrjn)

[Macronuclear DNA isolation and Illumina DNA-sequencing for IES retention score and alternative excision analyses 6](#_heading=h.26in1rg)

[Reference genomes and predicted genes 7](#_heading=h.35nkun2)

[IES retention and alternative boundary analysis 7](#_heading=h.1ksv4uv)

[Nucleosomal DNA Isolation and Illumina DNAse-sequencing 8](#_heading=h.44sinio)

[Nucleosome Density Analysis 9](#_heading=h.2jxsxqh)

[sRNA analysis 11](#_heading=h.z337ya)

[**Supplemental References 12**](#_heading=h.3j2qqm3)

# Supplemental Methods

## Culture Cultivation

Mating type 7 cells (strain 51) of *Paramecium tetraurelia* were grown according to the standard protocol [(Beisson et al. 2010b, 2010a)](https://sciwheel.com/work/citation?ids=6547099,9357048&pre=&pre=&suf=&suf=&sa=0,0). *E. coli* strain HT115 was used for feeding, and the cultures were maintained either at 27 °C or at 18 °C.

## RNAi assays

ICOPa and ICOPb RNAi constructs were made by cloning a 538 bp (1650-2188 in the coding sequence corresponding to the protein PTET.51.1.P0440186 in ParameciumDB) and a 1089 bp gene fragment (1130-2219 in the coding sequence corresponding to the protein PTET.51.1.P0180124 from ParameciumDB), respectively, into the L4440 plasmid. The plasmids were transformed into *E*. *coli* HT1115 (DE3). The ISWI1 RNAi construct is described in Singh et. al. 2022. Nowacki lab at the University of Bern, Switzerland, kindly provided DCL2, DCL3, PTIWI01 and PTIWI09 RNAi plasmids. Knockdown experiments were performed as previously [(Beisson et al. 2010c)](https://sciwheel.com/work/citation?ids=8105610&pre=&suf=&sa=0). Isopropyl ß-D-1-thiogalactopyranoside (IPTG) induction was done at 30 °C. Since starvation induces autogamy in *Paramecium*, we consider starved vegetative cells as T0. Cells were collected at early (T6-10: ~40% of cells with a fragmented parental MAC) and late (T12-T16: ~90% of cells with a visible new MAC) developmental time points. After three days, 30 post-autogamous cells were fed with a non-induced feeding medium to assay survival. Genomic DNA was extracted from the cultures that have completed MAC development using the standard kit protocol (G1N350, Sigma-Aldrich). PCRs were done on different genomic regions flanking an IES (Supplemental Table S5) to test IES retention.

## HHpred identification of protein domains

ICOPa (XM_001447768.1, PTET.51.1.P0440186) and ICOPb (XM_001437312.1, XM_001437313.1, PTET.51.1.P0180124) protein sequences were analyzed using HHpred from the MPI bioinformatics toolkit (<https://toolkit.tuebingen.mpg.de/#/>) across the COG [(Tatusov et al. 2000)](https://sciwheel.com/work/citation?ids=916911&pre=&suf=&sa=0), Pfam [(Finn et al. 2003, 2016)](https://sciwheel.com/work/citation?ids=1876155,1606475&pre=&pre=&suf=&suf=&sa=0,0), NCBI Conserved Domain Database (CDD) [(Marchler-Bauer et al. 2017)](https://sciwheel.com/work/citation?ids=3437700&pre=&suf=&sa=0), and ECOD [(Cheng et al. 2014, 2015)](https://sciwheel.com/work/citation?ids=1449747,178962&pre=&pre=&suf=&suf=&sa=0,0) databases. PSI-BLAST [(Bhagwat and Aravind 2007)](https://sciwheel.com/work/citation?ids=179387&pre=&suf=&sa=0) with 4 iterations was used to identify further proteins with WSD domains, and multiple alignments were done using MAFFT [(Katoh and Standley 2013; Kuraku et al. 2013)](https://sciwheel.com/work/citation?ids=387873,4237532&pre=&pre=&suf=&suf=&sa=0,0) provided as a plugin within Geneious (version 2022.1.1, http://www.geneious.com) [(Kearse et al. 2012)](https://sciwheel.com/work/citation?ids=459578&pre=&suf=&sa=0). InterProScan [(Paysan-Lafosse et al. 2023)](https://sciwheel.com/work/citation?ids=13949147&pre=&suf=&sa=0) within Geneious was used to identify domains within MAFFT-aligned protein sequences. For Fig. 1C Clustal Omega (1.2.3) from Geneious was used (default parameters). Proteins in the alignment are ICOPa (this study), ICOPb (this study), IOC3 [(Yamada et al. 2011)](https://sciwheel.com/work/citation?ids=1371658&pre=&suf=&sa=0), ACF [(Ito et al. 1997)](https://sciwheel.com/work/citation?ids=829386&pre=&suf=&sa=0), BAZ1B [(Bozhenok et al. 2002)](https://sciwheel.com/work/citation?ids=2084298&pre=&suf=&sa=0), DDW1 [(Yamada et al. 2011; Tan et al. 2020)](https://sciwheel.com/work/citation?ids=15495622,1371658&pre=&pre=&suf=&suf=&sa=0,0).

## Phylogenetic analysis of ICOPa and ICOPb

Trimal-auto [(Capella-Gutiérrez et al. 2009)](https://sciwheel.com/work/citation?ids=42475&pre=&suf=&sa=0) was used to select well-aligned columns from the MAFFT-aligned protein sequences of PSI-BLAST identified (see HHpred of protein domains) proteins. PHYML version 2.2.4 [(Guindon and Gascuel 2003)](https://sciwheel.com/work/citation?ids=178732&pre=&suf=&sa=0) provided as a plugin in Geneious (version 2022.1.1, http://www.geneious.com), was used to generate a maximum likelihood phylogeny with 100 bootstrap replicates. FigTree v1.4.4 (http://tree.bio.ed.ac.uk/software/figtree/) was used to inspect, manipulate and generate the graphical tree.

## Mass spectrometry analysis

Samples were separated on a 4%–12% NOVEX NuPage gradient gel (Thermo) for 10 minutes at 180 V in 1 X MES buffer (Thermo). Proteins were fixed and stained with Coomassie G250 brilliant blue (Carl Roth). The gel lanes were cut, and each lane was minced into approximately 1x1 mm pieces. Gel pieces were destained with a 50% ethanol/50 mM ammonium bicarbonate (ABC) solution. Proteins were reduced in 10 mM DTT (Sigma-Aldrich) for 1 hour at 56 °C and then alkylated with 50 mM iodoacetamide (Sigma-Aldrich) for 45 min at room temperature. Proteins were digested with 1 µg mass spectrometry grade trypsin (Sigma) overnight at 37 °C. Peptides were extracted from the gel by two incubations with 30% ABC/acetonitrile and three subsequent incubations with pure acetonitrile. The acetonitrile was subsequently evaporated in a concentrator (Eppendorf) and loaded on StageTips [(Rappsilber et al. 2007)](https://sciwheel.com/work/citation?ids=487704&pre=&suf=&sa=0) for desalting and storage.

Peptides were eluted from the StageTips using 80% acetonitrile / 0.1% formic acid and concentrated before loading on an uHPLC nLC-1200 system coupled to an Exploris 480 mass spectrometer (Thermo). The peptides were loaded on a 50 cm (Exploris 480) column (75 μm inner diameter) in-house packed with Reprosil C18 (Dr. Maisch GmbH) and eluted with a 73 or 88 min optimized gradient increasing from 3% to 40% mixture of 80% acetonitrile/0.1% formic acid at a flow rate of 225 nl/min or 250 nl/min. The Exploris 480 was operated in positive ion mode with a data-dependent acquisition strategy of one MS full scan (scan range 300 - 1,650 m/z; 60,000 resolution; normalized AGC target 300%; max IT 28 ms) and up to twenty MS/MS scans (15,000 resolution; AGC target 100%, max IT 40 ms; isolation window 1.4 m/z) with peptide match preferred using HCD fragmentation.

MS raw data were searched using the Andromeda search engine [(Cox et al. 2011)](https://sciwheel.com/work/citation?ids=921288&pre=&suf=&sa=0) integrated into MaxQuant suite 1.6.5.0 [(Cox and Mann 2008)](https://sciwheel.com/work/citation?ids=317588&pre=&suf=&sa=0) using the *Paramecium* predicted proteins as the database (ptetraurelia_mac_51_annotation_v2.0). In all analyses, carbamidomethylation at cysteine was set as a fixed modification, while methionine oxidation and protein N-acetylation were considered as variable modifications. The match between run option was activated. Prior to bioinformatics analysis, reverse hits, proteins only identified by site, protein groups based on one unique peptide, and known contaminants were removed.

For further bioinformatics analysis, the label-free quantitation (LFQ) values were log_2_ transformed, and the median across the replicates was calculated. This enrichment was plotted against the – log_10_ transformed p-value (Welch t-test) using the ggplot2 package in the R environment.

## Plasmids and vectors for recombinant protein expression assay

DNA sequences coding for *Paramecium* proteins ISWI1, ICOPa, and ICOPb were codon-optimized (Supplemental Table S9) for expression in *E. coli* using the GENEius tool of Eurofins (Luxembourg). Gene synthesis was performed at Eurofins Genomics Germany GmbH (Ebersberg, Germany). The synthetic constructs were cloned into pET-MCN vectors [(Romier et al. 2006)](https://sciwheel.com/work/citation?ids=1763191&pre=&suf=&sa=0), expressing proteins with either no tag, a hexahistidine (His), or a GST tag. Plasmids were co-transformed in different combinations into *E. coli* strain Gold pLysS.

## Total RNA extraction and mRNA sequencing.

Approximately 1.2 × 10^6^ cells were collected from the early (approx. 40% of cells with a fragmented MAC) and the late (majority of cells with visible anlagen) developmental stages using an oil centrifuge at 280 g for 2 minutes. The cells were washed twice in 10 mM Tris-HCl (pH 7.4), flash frozen using liquid nitrogen dropped gently over the pellets, and stored at -80°C. Total RNA extraction was performed by adding 6 ml of Tri reagent (Sigma-Aldrich, T9424) per sample and following the standard protocol provided with the reagent. mRNA and sRNA libraries were prepared and sequenced at Genewiz International (Leipzig, Germany).

## Knockdown efficiency validation using RNA-seq

Total RNA was sequenced by Genewiz (Germany, GmbH) using poly-A enrichment with NovaSeq 2×150 bp reads. Illumina adapter sequences were trimmed from reads with TrimGalore [(Krueger 2019)](https://sciwheel.com/work/citation?ids=15003635&pre=&suf=&sa=0) (Supplemental Table S6). Reads were mapped to the *Paramecium tetraurelia* strain 51 transcriptome. Mapping showed high coverage on the silencing regions, most likely caused by RNAs of the siRNA silencing pathway. For each knockdown, the target gene was replaced by three split transcripts (the silencing region, the 5’ upstream non-silencing region and the 3’ downstream non-silencing region), and only the 5’ upstream region was considered for analysis. FPKM (fragments per kilobase transcript per million mapped reads) values were calculated using eXpress [(Roberts and Pachter 2013)](https://sciwheel.com/work/citation?ids=586766&pre=&suf=&sa=0) (available from Edmond, the Max Planck Open Research Data repository, SourceData_Fig4; [(Singh 2023)](https://sciwheel.com/work/citation?ids=15193371&pre=&suf=&sa=0)) and rounded by the standard Python method to integers. Scripts are available from <https://github.com/Swart-lab/ICOP_code/tree/main/KD-efficiency>.

## Macronuclear DNA isolation and Illumina DNA-sequencing for IES retention score and alternative excision analyses

MAC DNA was purified from *ICOPa-KD, ICOPb-*KD*,* and *ICOPa/b-*KD cultures three days post-development, as described previously [(Arnaiz et al. 2012)](https://sciwheel.com/work/citation?ids=2201947&pre=&suf=&sa=0). DNA libraries were prepared using the FS DNA Library Prep kit (E7805, NEB). Paired-end (2×150 bp) sequencing was done on NextSeq 2000 at MPI for Biology, Tübingen.

##

## Reference genomes and predicted genes

Reference genome to analyze DNA-seq data:

MAC: <https://paramecium.i2bc.paris-saclay.fr/files/Paramecium/tetraurelia/51/sequences/ptetraurelia_mac_51.fa>

MAC+IES: <https://paramecium.i2bc.paris-saclay.fr/files/Paramecium/tetraurelia/51/sequences/ptetraurelia_mac_51_with_ies.fa>

Reference CDS + UTR sequences used to analyze mRNA-seq data:

<https://paramecium.i2bc.paris-saclay.fr/files/Paramecium/tetraurelia/51/annotations/ptetraurelia_mac_51/ptetraurelia_mac_51_annotation_v2.0.transcript.fa>

## IES retention and alternative boundary analysis

Illumina adapter sequences were trimmed from whole genome sequencing (WGS) reads of enriched new MAC DNA after knockdown using TrimGalore [(Krueger 2019)](https://sciwheel.com/work/citation?ids=15003635&pre=&suf=&sa=0) (Supplemental Table S6). ParTIES [(Denby Wilkes et al. 2016)](https://sciwheel.com/work/citation?ids=4034499&pre=&suf=&sa=0) v1.05 was used to map reads to MAC and MAC+IES genomes and calculate IRSs.

To accommodate changes in a newer version of samtools [(Li et al. 2009)](https://sciwheel.com/work/citation?ids=48787&pre=&suf=&sa=0), the /lib/PARTIES/Map.pm file was changed (Supplemental Table S7). IRSs are provided at Edmond, the Max Planck Open Research Data repository, SourceData_Fig4 [(Singh 2023)](https://sciwheel.com/work/citation?ids=15193371&pre=&suf=&sa=0) as ICOP_IRS.tab.gz. IRS correlations using IRSs from published knockdown data ((*ISWI1*-KD [(Singh et al. 2022)](https://sciwheel.com/work/citation?ids=13753998&pre=&suf=&sa=0), *PGM*-KD [(Arnaiz et al. 2012)](https://sciwheel.com/work/citation?ids=2201947&pre=&suf=&sa=0), *TFIIS4*-KD [(Maliszewska-Olejniczak et al. 2015)](https://sciwheel.com/work/citation?ids=4034484&pre=&suf=&sa=0) and *PTIWI01/09*-KD [(Furrer et al. 2017)](https://sciwheel.com/work/citation?ids=4034503&pre=&suf=&sa=0)) were calculated with After_ParTIES (option --use_pearson (<https://github.com/gh-ecs/After_ParTIES>)).

Since alternative excision analysis depends on IES coverage, to ensure a fair comparison, libraries were adjusted to similar sizes by downsampling. Downsampling factors relative to the smallest library used were calculated according to the number of properly paired and mapped reads to the MAC+IES reference genome (ND7 = 0.686; ICOPa = 0.512; ICOPb = 0.453; ISWI1 = 0.698; ICOPa_b = 1.0). The “MILORD” module of a ParTIES pre-release version (13 August 2015) was used to annotate alternative and cryptic IES excision (annotations available from Edmond, the Max Planck Open Research Data repository, SourceData_Fig5; [(Singh 2023)](https://sciwheel.com/work/citation?ids=15193371&pre=&suf=&sa=0)).

All scripts are available from <https://github.com/Swart-lab/ICOP_code/tree/main/Alternative_excision>.

## Nucleosomal DNA Isolation, Illumina DNAse-sequencing and associated DNA-seq

Nucleosomal DNA was isolated with the EZ Nucleosomal DNA Prep Kit (D5220, Zymo Research) as previously utilizing a sucrose cushion to isolate nuclei [(Singh et al. 2022)](https://sciwheel.com/work/citation?ids=13753998&pre=&suf=&sa=0). *ICOPa/b/PGM*-KD and *ND7/PGM*-KD cultures were collected ca. 12 h after developing MACs were visible in most cells. We split the nuclei into two samples, then used the undigested samples for MAC DNA-seq and DNAse digested samples for DNAse-seq. DNA-seq libraries were prepared as mentioned in the section above using FS DNA Library Prep kit (E7805, NEB). For the DNAse-seq libraries the digested DNA was size-selected with SPRIselect magnetic beads (Beckman Coulter) to enrich mono- and di-nucleosomal fragments (0.7× volume right-side size selection). Libraries were prepared with NEBNext Ultra II DNA library prep kit (E7645S, NEB), size-selected for 150 bp insert. 2×100 bp paired-end sequencing was performed on an Illumina NextSeq 2000 instrument with P3 chemistry at MPI for Biology, Tübingen.

## Nucleosome Density Analysis

Illumina adapter sequences were trimmed from reads with TrimGalore [(Krueger 2019)](https://sciwheel.com/work/citation?ids=15003635&pre=&suf=&sa=0) (Supplemental Table S6).

Reads were mapped to the MAC+IES genome, then properly paired and mapped reads overlapping IESs were extracted and counted. IRS distributions are provided in Supplemental Fig. 7A. DNase reads were size selected (100 - 175 bp outer distance). Library sizes to calculate downsampling factors were obtained by the “samtools stats” command on the .sorted.bam files. The length distribution of outer distances of PE reads mapping to scaffold51_9 was plotted (Supplemental Fig. 7B).

As previously (Singh et al. 2022):

d_c_ = number of DNA reads mapped to a particular IES from control.

n_c_ = number of nucleosomal reads mapped to a particular IES from control.

d_e_ = number of DNA reads mapped to a particular IES from experiment.

n_e_ = number of nucleosomal reads mapped to a particular IES from experiment.

D_c_ = sum(d_c_).

N_c_ = sum(n_c_).

D_e_ = sum(d_e_).

N_e_ = sum(n_e_).

r_c_=(n_c_/N_c_)÷(d_c_/D_c_)

r_e_=(n_e_/N_e_)÷(d_e_/D_e_).

Samples used for nucleosome density analysis are in Supplemental Table S10. Nucleosome density differences (re_rc) were calculated for each IES by subtracting the nucleosome density of the control (r_c_) from the experimental sample (r_e_). In Jupyter notebook notation without subscripts:

re_rc = r_e - r_c

IES with infinite (“inf”) or not available “nan” values were excluded, resulting in 43,409 (in *NOWA1/2/PGM*-KD) and 44,448 (in *ICOPa/b/PGM*-KD) IESs used for analysis. Kolmogorov-Smirnov (KS) statistics and associated p-values for two sample tests were calculated to assess distribution differences.

All scripts are available from <https://github.com/Swart-lab/ICOP_code/tree/main/Nucleosome_density>.

IES read counts from htseq-count are available from Edmond, the Max Planck Open Research Data repository, “SourceData_Fig7” [(Singh 2023)](https://sciwheel.com/work/citation?ids=15193371&pre=&suf=&sa=0).

## sRNA analysis

sRNA-seq reads were mapped to the *Paramecium tetraurelia* strain 51 MAC + IES genome and L4440 silencing vector with bwa version 0.7.17-r1188 [(Li and Durbin 2009)](https://sciwheel.com/work/citation?ids=48641&pre=&suf=&sa=0). 10-49 bp long, uniquely mapped reads (possessing the flags “XT:A:U”) were selected by grep in a shell script. sRNA length histograms were generated by a Python script. Shell scripts for the RNA mapping, post-processing, and histogram are available from <https://github.com/Swart-lab/ICOP_code/tree/main/sRNA_analysis>.

# Supplemental References

[Arnaiz O, Mathy N, Baudry C, Malinsky S, Aury J-M, Denby Wilkes C, Garnier O, Labadie K, Lauderdale BE, Le Mouël A, et al. 2012. The *Paramecium* germline genome provides a niche for intragenic parasitic DNA: evolutionary dynamics of internal eliminated sequences. *PLoS Genet* **8**: e1002984.](https://sciwheel.com/work/bibliography/2201947)

[Beisson J, Bétermier M, Bré M-H, Cohen J, Duharcourt S, Duret L, Kung C, Malinsky S, Meyer E, Preer JR, et al. 2010a. Maintaining clonal *Paramecium tetraurelia* cell lines of controlled age through daily reisolation. *Cold Spring Harb Protoc* **2010**: pdb.prot5361.](https://sciwheel.com/work/bibliography/9357048)

[Beisson J, Bétermier M, Bré M-H, Cohen J, Duharcourt S, Duret L, Kung C, Malinsky S, Meyer E, Preer JR, et al. 2010b. Mass culture of *Paramecium tetraurelia*. *Cold Spring Harb Protoc* **2010**: pdb.prot5362.](https://sciwheel.com/work/bibliography/6547099)

[Beisson J, Bétermier M, Bré M-H, Cohen J, Duharcourt S, Duret L, Kung C, Malinsky S, Meyer E, Preer JR, et al. 2010c. Silencing specific Paramecium tetraurelia genes by feeding double-stranded RNA. *Cold Spring Harb Protoc* **2010**: pdb.prot5363.](https://sciwheel.com/work/bibliography/8105610)

[Bhagwat M, Aravind L. 2007. PSI-BLAST tutorial. *Methods Mol Biol* **395**: 177–186.](https://sciwheel.com/work/bibliography/179387)

[Bozhenok L, Wade PA, Varga-Weisz P. 2002. WSTF-ISWI chromatin remodeling complex targets heterochromatic replication foci. *EMBO J* **21**: 2231–2241.](https://sciwheel.com/work/bibliography/2084298)

[Capella-Gutiérrez S, Silla-Martínez JM, Gabaldón T. 2009. trimAl: a tool for automated alignment trimming in large-scale phylogenetic analyses. *Bioinformatics* **25**: 1972–1973.](https://sciwheel.com/work/bibliography/42475)

[Cheng H, Liao Y, Schaeffer RD, Grishin NV. 2015. Manual classification strategies in the ECOD database. *Proteins* **83**: 1238–1251.](https://sciwheel.com/work/bibliography/178962)

[Cheng H, Schaeffer RD, Liao Y, Kinch LN, Pei J, Shi S, Kim B-H, Grishin NV. 2014. ECOD: an evolutionary classification of protein domains. *PLoS Comput Biol* **10**: e1003926.](https://sciwheel.com/work/bibliography/1449747)

[Cox J, Mann M. 2008. MaxQuant enables high peptide identification rates, individualized p.p.b.-range mass accuracies and proteome-wide protein quantification. *Nat Biotechnol* **26**: 1367–1372.](https://sciwheel.com/work/bibliography/317588)

[Cox J, Neuhauser N, Michalski A, Scheltema RA, Olsen JV, Mann M. 2011. Andromeda: a peptide search engine integrated into the MaxQuant environment. *J Proteome Res* **10**: 1794–1805.](https://sciwheel.com/work/bibliography/921288)

[Denby Wilkes C, Arnaiz O, Sperling L. 2016. ParTIES: a toolbox for *Paramecium* interspersed DNA elimination studies. *Bioinformatics* **32**: 599–601.](https://sciwheel.com/work/bibliography/4034499)

[Finn R, Griffiths-Jones S, Bateman A. 2003. Identifying protein domains with the Pfam database. *Curr Protoc Bioinformatics* **Chapter 2**: Unit 2.5.](https://sciwheel.com/work/bibliography/1606475)

[Finn RD, Coggill P, Eberhardt RY, Eddy SR, Mistry J, Mitchell AL, Potter SC, Punta M, Qureshi M, Sangrador-Vegas A, et al. 2016. The Pfam protein families database: towards a more sustainable future. *Nucleic Acids Res* **44**: D279-85.](https://sciwheel.com/work/bibliography/1876155)

[Furrer DI, Swart EC, Kraft MF, Sandoval PY, Nowacki M. 2017. Two Sets of Piwi Proteins Are Involved in Distinct sRNA Pathways Leading to Elimination of Germline-Specific DNA. *Cell Rep* **20**: 505–520.](https://sciwheel.com/work/bibliography/4034503)

[Guindon S, Gascuel O. 2003. A simple, fast, and accurate algorithm to estimate large phylogenies by maximum likelihood. *Syst Biol* **52**: 696–704.](https://sciwheel.com/work/bibliography/178732)

[Ito T, Bulger M, Pazin MJ, Kobayashi R, Kadonaga JT. 1997. ACF, an ISWI-containing and ATP-utilizing chromatin assembly and remodeling factor. *Cell* **90**: 145–155.](https://sciwheel.com/work/bibliography/829386)

[Katoh K, Standley DM. 2013. MAFFT multiple sequence alignment software version 7: improvements in performance and usability. *Mol Biol Evol* **30**: 772–780.](https://sciwheel.com/work/bibliography/387873)

[Kearse M, Moir R, Wilson A, Stones-Havas S, Cheung M, Sturrock S, Buxton S, Cooper A, Markowitz S, Duran C, et al. 2012. Geneious Basic: an integrated and extendable desktop software platform for the organization and analysis of sequence data. *Bioinformatics* **28**: 1647–1649.](https://sciwheel.com/work/bibliography/459578)

[Krueger F. 2019. *TrimGalore: A wrapper around Cutadapt and FastQC to consistently apply adapter and quality trimming to FastQ files, with extra functionality for RRBS data*. GitHub.](https://sciwheel.com/work/bibliography/15003635)

[Kuraku S, Zmasek CM, Nishimura O, Katoh K. 2013. aLeaves facilitates on-demand exploration of metazoan gene family trees on MAFFT sequence alignment server with enhanced interactivity. *Nucleic Acids Res* **41**: W22-8.](https://sciwheel.com/work/bibliography/4237532)

[Li H, Durbin R. 2009. Fast and accurate short read alignment with Burrows-Wheeler transform. *Bioinformatics* **25**: 1754–1760.](https://sciwheel.com/work/bibliography/48641)

[Li H, Handsaker B, Wysoker A, Fennell T, Ruan J, Homer N, Marth G, Abecasis G, Durbin R, 1000 Genome Project Data Processing Subgroup. 2009. The Sequence Alignment/Map format and SAMtools. *Bioinformatics* **25**: 2078–2079.](https://sciwheel.com/work/bibliography/48787)

[Maliszewska-Olejniczak K, Gruchota J, Gromadka R, Denby Wilkes C, Arnaiz O, Mathy N, Duharcourt S, Bétermier M, Nowak JK. 2015. TFIIS-Dependent Non-coding Transcription Regulates Developmental Genome Rearrangements. *PLoS Genet* **11**: e1005383.](https://sciwheel.com/work/bibliography/4034484)

[Marchler-Bauer A, Bo Y, Han L, He J, Lanczycki CJ, Lu S, Chitsaz F, Derbyshire MK, Geer RC, Gonzales NR, et al. 2017. CDD/SPARCLE: functional classification of proteins via subfamily domain architectures. *Nucleic Acids Res* **45**: D200–D203.](https://sciwheel.com/work/bibliography/3437700)

[Paysan-Lafosse T, Blum M, Chuguransky S, Grego T, Pinto BL, Salazar GA, Bileschi ML, Bork P, Bridge A, Colwell L, et al. 2023. InterPro in 2022. *Nucleic Acids Res* **51**: D418–D427.](https://sciwheel.com/work/bibliography/13949147)

[Rappsilber J, Mann M, Ishihama Y. 2007. Protocol for micro-purification, enrichment, pre-fractionation and storage of peptides for proteomics using StageTips. *Nat Protoc* **2**: 1896–1906.](https://sciwheel.com/work/bibliography/487704)

[Roberts A, Pachter L. 2013. Streaming fragment assignment for real-time analysis of sequencing experiments. *Nat Methods* **10**: 71–73.](https://sciwheel.com/work/bibliography/586766)

[Romier C, Ben Jelloul M, Albeck S, Buchwald G, Busso D, Celie PHN, Christodoulou E, De Marco V, van Gerwen S, Knipscheer P, et al. 2006. Co-expression of protein complexes in prokaryotic and eukaryotic hosts: experimental procedures, database tracking and case studies. *Acta Crystallogr D Biol Crystallogr* **62**: 1232–1242.](https://sciwheel.com/work/bibliography/1763191)

[Singh A, Maurer-Alcalá XX, Solberg T, Häußermann L, Gisler S, Ignarski M, Swart EC, Nowacki M. 2022. Chromatin remodeling is required for sRNA-guided DNA elimination in Paramecium. *EMBO J* **41**: e111839.](https://sciwheel.com/work/bibliography/13753998)

[Singh A. 2023. ISWI complex proteins facilitate genome editing and development. *Edmond*. https://doi.org/10.17617/3.ZBOLU8 (Accessed July 28, 2023).](https://sciwheel.com/work/bibliography/15193371)

[Tan L-M, Liu R, Gu B-W, Zhang C-J, Luo J, Guo J, Wang Y, Chen L, Du X, Li S, et al. 2020. Dual recognition of h3k4me3 and DNA by the ISWI component ARID5 regulates the floral transition in arabidopsis. *Plant Cell* **32**: 2178–2195.](https://sciwheel.com/work/bibliography/15495622)

[Tatusov RL, Galperin MY, Natale DA, Koonin EV. 2000. The COG database: a tool for genome-scale analysis of protein functions and evolution. *Nucleic Acids Res* **28**: 33–36.](https://sciwheel.com/work/bibliography/916911)

[Wang C, Solberg T, Maurer-Alcalá XX, Swart EC, Gao F, Nowacki M. 2022. A small RNA-guided PRC2 complex eliminates DNA as an extreme form of transposon silencing. *Cell Rep* **40**: 111263.](https://sciwheel.com/work/bibliography/13513385)

[Yamada K, Frouws TD, Angst B, Fitzgerald DJ, DeLuca C, Schimmele K, Sargent DF, Richmond TJ. 2011. Structure and mechanism of the chromatin remodelling factor ISW1a. *Nature* **472**: 448–453.](https://sciwheel.com/work/bibliography/1371658)
